# βPix-d promotes tubulin acetylation and neurite outgrowth through a PAK/Stathmin1 signaling pathway

**DOI:** 10.1371/journal.pone.0230814

**Published:** 2020-04-06

**Authors:** Younghee Kwon, Ye Won Jeon, Minjae Kwon, Yongcheol Cho, Dongeun Park, Jung Eun Shin

**Affiliations:** 1 School of Biological Sciences, Seoul National University, Seoul, Republic of Korea; 2 Division of Life Sciences, College of Life Sciences and Biotechnology, Korea University, Seoul, Republic of Korea; 3 Institute of Life Science and Biotechnology, Korea University, Seoul, Republic of Korea; NCMLS, Radboud University Nijmegen Medical Center, NETHERLANDS

## Abstract

Microtubules are a major cytoskeletal component of neurites, and the regulation of microtubule stability is essential for neurite morphogenesis. βPix (*ARHGEF7*) is a guanine nucleotide exchange factor for the small GTPases Rac1 and Cdc42, which modulate the organization of actin filaments and microtubules. βPix is expressed as alternatively spliced variants, including the ubiquitous isoform βPix-a and the neuronal isoforms βPix-b and βPix-d, but the function of the neuronal isoforms remains unclear. Here, we reveal the novel role of βPix neuronal isoforms in regulating tubulin acetylation and neurite outgrowth. At DIV4, hippocampal neurons cultured from βPix neuronal isoform knockout (βPix-NIKO) mice exhibit defects in neurite morphology and tubulin acetylation, a type of tubulin modification which often labels stable microtubules. Treating βPix-NIKO neurons with paclitaxel, which stabilizes the microtubules, or reintroducing either neuronal βPix isoform to the KO neurons overcomes the impairment in neurite morphology and tubulin acetylation, suggesting that neuronal βPix isoforms may promote microtubule stabilization during neurite development. βPix-NIKO neurons also exhibit lower phosphorylation levels for Stathmin1, a microtubule-destabilizing protein, at Ser16. Expressing either βPix neuronal isoform in the βPix-NIKO neurons restores Stathmin1 phosphorylation levels, with βPix-d having a greater effect than βPix-b. Furthermore, we find that the recovery of neurite length and Stathmin1 phosphorylation via βPix-d expression requires PAK kinase activity. Taken together, our study demonstrates that βPix-d regulates the phosphorylation of Stathmin1 in a PAK-dependent manner and that neuronal βPix isoforms promote tubulin acetylation and neurite morphogenesis during neuronal development.

## Introduction

Neural development requires the neuronal morphogenesis, during which the formation and outgrowth of neurites are supported by the coordinated reorganization of actin and microtubules [1]. Microtubules are major components of the cytoskeleton in dendritic and axonal shafts [2]. Post-translational modifications of the microtubules vary in different regions of a neuron and change during neuronal morphogenesis. Those modifications affect the dynamics and stability of the microtubules that contribute to neuronal morphogenesis [3, 4]. The deregulation of microtubule dynamics via genetic or pharmacological manipulation of the tubulin post-translational modifications often leads to defective neurite morphogenesis [4–7]. Impaired microtubule stability has been consistently observed in many neurodevelopmental disorders such as intellectual disabilities and autism spectrum disorder [8].

Stathmin1 is a cytosolic phosphoprotein that functions as a microtubule-destabilizing factor. Stathmin1 destabilizes microtubules by sequestration of α- and β-tubulin heterodimers, inhibiting microtubule polymerization and promoting microtubule catastrophe [9, 10]. The microtubule-destabilizing activity of Stathmin1 is suppressed by phosphorylation at four serine (Ser) sites (Ser16, Ser25, Ser38, and Ser63), which reduces its affinity for tubulin dimers and consequently promotes microtubule assembly. Phosphorylation at Ser16 or Ser63 is known to be more critical for the inactivation of Stathmin1 *in vitro* than that at Ser25 or Ser38 [11–15]. In neurons, Stathmin1 regulates the development of axons [16] and dendrites [17] via phosphorylation at Ser16, with p21-activated kinase (PAK), a kinase activated by the small GTPases Rac1 and Cdc42 [18], responsible for Stathmin1 phosphorylation at this site [19].

**Fig 1:**
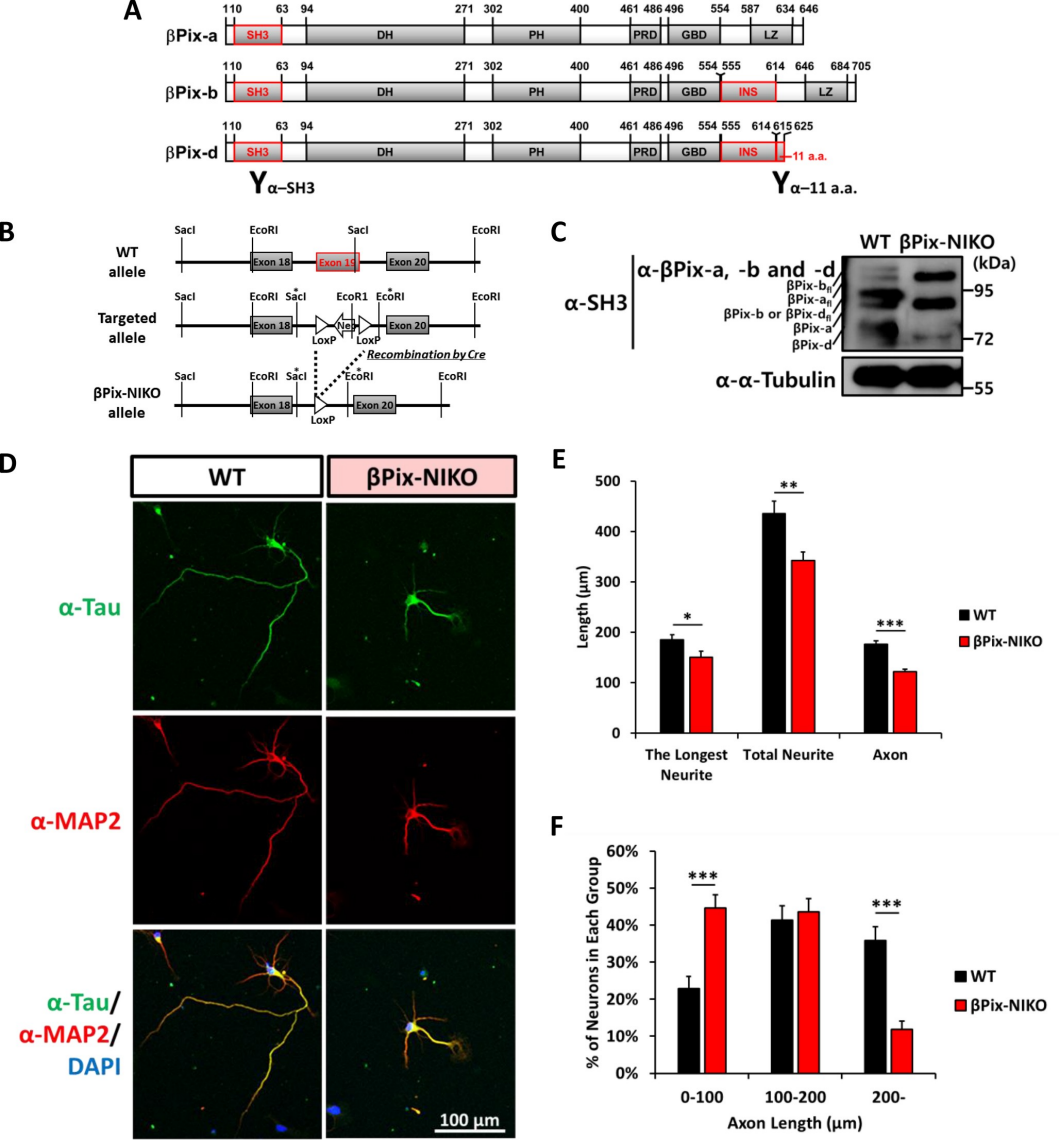
The outgrowth and branching of neurites and axons are impaired in hippocampal neurons from βPix neuronal isoform KO (βPix-NIKO) mice. (A) Domain structure of βPix-a, βPix-b, and βPix-d isoforms. SH3 = Src homology 3 domain, DH = Dbl homology domain, PH = Pleckstrin homology domain, PRD = Proline-rich domain, GBD = GIT1-binding domain, LZ = Leucine zipper domain, INS = novel insert region, 11 a.a. = addition of 11 amino acids region. The INS domain is specific for the neuronal isoforms βPix-b and βPix-d and the 11 a.a. region is specific for βPix-d isoform. Antibodies against the SH3 and 11 a.a. domains are indicated. (B) Gene targeting strategy for βPix-NIKO mice. Exon 19 (red box) encodes the neuronal isoform-specific INS domain. Expression of the neuronal isoforms is constitutively eliminated by targeting exon 19 by a neomycin-resistance gene cassette and subsequently removing the cassette by Cre-based recombination. (C) Expression patterns of βPix-a, βPix-b, and βPix-d in hippocampal neurons from WT and βPix-NIKO mice at DIV4. βPix-a_fl_, βPix-b_fl,_ and βPix-d_fl_ indicate the full-length versions of individual βPix splicing variants that harbor additional 5’ exons. Expression of the ubiquitous βPix-a isoform is preserved in the βPix-NIKO neurons. (D) Representative images of hippocampal neurons cultured from WT and βPix-NIKO mice fixed at DIV4 and stained with Tau antibody (green), MAP2 antibody (red), and DAPI (blue). (E) Analysis results for the length of the neurites and axons. The βPix-NIKO neurons show a 19% decrease in the longest neurite length, a 21% decrease in total neurite length, and a 31% decrease in average axon length compared with the WT neurons. (F) The βPix-NIKO cultures have more neurons with axons shorter than 100 μm and fewer neurons with axons longer than 200 μm compared to cultures from WT mice. n = 53–58 neurons per group for the longest neurite and total neurite assays in (E) and n = 162–195 axons per group for the axon assays in (E) and (F) from three independent cultures. * *P* < 0.05, ** *P* < 0.01, and *** *P* < 0.001 for the comparison of WT and βPix-NIKO neurons by Student’s *t*-tests.

Beta-PAK interacting exchange factor (βPix) acts as a guanine nucleotide exchange factor (GEF) that specifically activates Rac1 and Cdc42 [20, 21] and also regulates the activity of PAK via direct binding [18, 22]. Rac1 and Cdc42 mediate different steps in neuronal morphogenesis, including neurite outgrowth and synapse formation, by regulating the reorganization of actin and microtubule [23]. Several studies have reported that βPix-a, a ubiquitous βPix isoform, regulates the formation of axons, spines, and synapses [24–28]. We have previously identified βPix-b and βPix-d, which are alternative spliced βPix isoforms that are specifically expressed in neurons [29, 30] (Fig 1A). Recent studies have found that βPix-b plays an essential role in dendritic spine morphogenesis [31, 32], while the neuronal role of βPix-d remains unclear.

In the present study, we demonstrate that βPix-d promotes neurite outgrowth by increasing tubulin acetylation. At 4 days *in vitro* (DIV4), hippocampal neurons cultured from βPix neuronal isoform knockout (βPix-NIKO) mice, in which the expression of βPix-b and βPix-d is specifically eliminated, exhibit reduced neurite length and tubulin acetylation. Treating the βPix-NIKO cultures with the microtubule-stabilizing agent paclitaxel suppresses the defects in neuronal morphology and tubulin acetylation, indicating that the morphological phenotype is the result of impaired microtubule stability. We identify βPix-d as the primary βPix isoform involved in tubulin acetylation because rescuing βPix-d expression in the βPix-NIKO cultures restores tubulin acetylation and neurite outgrowth to a greater extent than βPix-b. We also find that Stathmin1 phosphorylation at Ser16 is impaired in βPix-NIKO neurons and that the subsequent expression of βPix-d is sufficient to restore Ser16 phosphorylation levels. By utilizing the expression of the PAK inhibitory domain (PID), we demonstrate that the role of βPix-d in Stathmin1 phosphorylation and neurite outgrowth is dependent on PAK activity. Taken together, our results show that βPix-d promotes neurite development via regulating microtubule stability and PAK-induced Stathmin1 phosphorylation at Ser16.

## Materials and methods

### Mice

To generate βPix-NIKO mice, exon 19 of *ARHGEF7*, the gene encoding the mouse βPix protein, was replaced with a neomycin-resistance cassette using a targeted knockout (KO) approach. The Cre-mediated excision of the neomycin-resistant gene flanked by LoxP sites was conducted *in vivo* via crossbreeding with mice harboring a Sox2 promotor-driven Cre transgene (Fig 1B). Because exon 19 is specific to the mRNA of the βPix-b and βPix-d isoforms, the excision only prevented the expression of these isoforms while preserving the expression of βPix-a (Fig 1C), resulting in a constitutive KO allele for the neuronal isoforms. Mice heterozygous for the KO allele were then interbred to produce homozygous KO mice. Animals were bred and kept at a constant 23˚C and 40–60% humidity in specific pathogen-free animal facilities at Seoul National University. All mice were housed by genotype with four or five mice per cage under a 12-h light/dark cycle with food and water available *ad libitum*. The Animal Research Committee at Seoul National University specifically approved all experiments conducted in this study (SNU-160321-2-5). Adult mice and neonates were euthanized by CO_2_ inhalation and decapitation, respectively.

### Reagents and primary antibodies

Paclitaxel was purchased from Sigma. To produce the polyclonal rabbit antibodies required to detect the βPix isoforms, the βPix SH3 domain fused to glutathione S-transferase (GST) was purified using a glutathione affinity column as described previously [26, 29]. Likewise, to create the antibodies for the detection of both βPix neuronal isoforms or βPix-d only, GST fusion proteins of the neuronal isoform-specific insert (INS) domain and 11 βPix-d-specific amino acids (a.a.) were generated and employed in immunization. The following commercially available antibodies were used for Western blot and immunostaining analysis: monoclonal mouse antibody against acetylated α-tubulin (clone 6-11B-1, Sigma), monoclonal rabbit antibody against acetylated α-tubulin (clone D20G3, Cell Signaling), monoclonal mouse antibody against GFP (clone B-2, Santa Cruz), polyclonal rabbit antibody against MAP2 (Cell Signaling), monoclonal mouse antibody against Myc (clone 9E10, Santa Cruz), monoclonal rabbit antibody against Stathmin1 (EP1573Y, Abcam), polyclonal rabbit antibody against Stathmin1 (phospho S16, Abcam), monoclonal mouse antibody against tyrosinated α-tubulin (clone TUB-1A2, Sigma), monoclonal mouse antibody against Tau (clone Tau-5, Chemicon), monoclonal mouse antibody against α-tubulin (clone DM1A, Abcam), and monoclonal mouse antibody against β3-tubulin (clone TU-20, Chemicon).

### Constructs

Expression vectors were cloned using a PCR-based approach into pEGFP-N1 (Clontech) and pcDNA3.1 myc/his (Invitrogen) vectors. To generate GFP-βPix-a, GFP-βPix-b, or GFP-βPix-d, the cDNA of βPix-a, βPix-b, or βPix-d was isolated from a mouse brain cDNA library [29, 30] and the coding region of βPix-a, βPix-b, or βPix-d was subcloned into pEGFP-N1 using PCR. To generate Myc-PID, the 83–149 a.a. of PAK1 coding regions (NM_001357363), which can inhibit all group I PAKs, were subcloned into a pcDNA3.1 myc/his vector using PCR [33].

### Western blot analysis

Cells were washed twice with phosphate-buffered saline (PBS) or tris-buffered saline (TBS) and lysed with SDS-lysis buffer (100 mM Tris, pH 6.8, 2% SDS, and 10% glycerol). The concentration of protein was determined using BCA reagent (Thermo Scientific). Equal amounts of total protein were resolved with SDS-PAGE and transferred to a polyvinylidine difluoride (Millipore) membrane. Blots were blocked with 3% bovine serum albumin in 0.1% Triton X-100 in PBS (0.1% PBS-T) or 0.1% Tween 20 in TBS (0.1% TBS-T) for 30 min. The blots were incubated with primary antibodies for 1 h at room temperature and washed with 0.1% PBS-T or 0.1% TBS-T. The blots were then incubated with horseradish peroxidase-conjugated secondary antibodies (Jackson ImmunoResearch Laboratories, Inc.) and analyzed using enhanced chemiluminescence reagents. Tubulin was used as a loading control.

### Primary hippocampal neuron culture and transfection

Mouse hippocampal cultures were prepared from postnatal day 0–1 mouse pups of either sex as previously described [34]. Dissociated hippocampus tissue was treated with papain (20 μg/ml) and DNase (10 units/μl) for 20 min at 37°C. The tissue was then mechanically dissociated using trituration with a Pasteur pipette. Hippocampal neurons (2 x 10^5^ cells / 60 mm dish) were plated in Minimum Essential Media (Welgene) supplemented with 0.6% glucose, 1 mM sodium pyruvate, 1% penicillin-streptomycin (Gibco), 2 mM L-glutamine, and 10% certified fetal bovine serum (c-FBS, Gibco) for 4 h before it was exchanged with Neurobasal Medium (Gibco) supplemented with 0.5 mM L-glutamine and B27 supplement (Gibco). The cells were maintained in a 5% CO_2_ incubator at 37°C. Every four to seven days, half of the original media was discarded and replenished with fresh Neurobasal Medium supplemented with 0.5 mM glutamine and B27 supplement. When the hippocampal neurons were transfected at DIV3, Lipofectamine 2000 (Invitrogen) was used according to the manufacturer’s instructions.

### Mouse Embryonic Fibroblast (MEF) culture and transfection

MEF culture and transfection were performed as described previously [35]. Briefly, MEFs were cultured in DMEM (Gibco) supplemented with 10% cFBS, 1% MEM non-essential amino acid (Gibco), 1% L-glutamine, 0.1% β-mercaptoethanol (Gibco) and 1% antibiotics/antimycotics mixture (Gibco) in 5% CO2 incubator at 37°C. Coverslips or dishes was coated with 10 μg/ml fibronectin. The MEFs were transfected with pEGFP-C1 with Metafectene Pro (Biontex Laboratories) for 24 h.

### Immunocytochemistry

DIV4 mouse hippocampal neurons and MEFs seeded on 12-mm coverslips were fixed in 3.7% paraformaldehyde in PBS or TBS for 10 min at room temperature. The neurons were permeabilized with 0.5% PBS-T or 0.5% TBS-T for 10 min and then incubated in blocking solution (10% c-FBS and 0.5% gelatin in 0.1% PBS-T or 0.1% TBS-T) for 30 min. The coverslips were then incubated with primary antibodies diluted in blocking solution for 1 h at room temperature. After washing with 0.1% PBS-T or 0.1% TBS-T, the coverslips were stained with fluorescein isothiocyanate (FITC)-conjugated or tetramethyl rhodamine isothiocyanate (TRITC)-conjugated anti-mouse or anti-rabbit IgG (Jackson ImmunoResearch Laboratories, Inc.), aminomethylcoumarin acetate (AMCA)-conjugated anti-rabbit IgG (Jackson ImmunoResearch Laboratories, Inc.), or Alexa Fluor 350-conjugated anti-mouse IgG (Invitrogen) for 1 h. For F-actin staining, the coverslips were stained with rhodamine phalloidin (Molecular Probes). Following incubation, the coverslips were washed with 0.1% PBS-T or 0.1% TBS-T and mounted with Vectashield (Vector Laboratories). To stain the nucleus, DAPI (Molecular Probes) was mixed with the mounting solution. The stained neurons and MEFs were observed with a Zeiss LSM700 confocal microscope equipped with a 20x, 0.8 Plan-Apochromat objective and a 40x, 1.20 C-Apochromat objective. Imaging settings were kept constant for all images in the same experiment and Z-stacked images were converted to maximal projection.

### Experimental design and statistical analysis

Immunofluorescent images of the cultured neurons were quantified using ImageJ software (NIH) in a blind manner and the measured values were transferred to Excel (Microsoft). The exact length of the neurites and axons was measured using ImageJ. For quantification of fluorescence intensity and neurite morphology, the longest neurite was analyzed, given that most of the longest neurite were stained with tau antibody. To visualize the distribution of fluorescence intensities, we performed a line scan with ImageJ. All data were expressed as the mean ± standard error. All analyses were conducted using a minimum of three independent experiments and statistically evaluated using Excel and SPSS (IBM). Statistical comparisons between groups were analyzed for significance by Student’s *t*-test or one-way ANOVA followed by post-hoc Tukey’s test, as specified in legend of each figure. The *P*-values are indicated in the figure and supporting information legends.

## Results

### Loss of neuronal βPix isoforms impairs neurite outgrowth and branching in cultured hippocampal neurons

Neurite elongation and branching are essential during neuronal development, and Rac1 and Cdc42 small GTPases, which are activated by βPix, play a pivotal role in neurite morphogenesis [36]. To investigate the dependence of neurite morphogenesis on neuronal βPix isoforms, we analyzed the morphology of hippocampal neurons cultured from WT and βPix-NIKO mice at DIV4, when axonal and dendritic outgrowth occurs. To assess the morphological features, we labeled the neurons with Tau antibody as an axonal marker and MAP2 antibody as a dendritic marker (Fig 1D). Notably, there was a significant decrease in the length of the longest neurite and total neurite length in the βPix-NIKO neurons when compared to WT neurons (Fig 1E), suggesting that βPix neuronal isoforms are required for neurite outgrowth. We found that the number of neurite branching points was also lower in the neurons cultured from βPix-NIKO mice than in WT neurons (WT, 2.70 ± 0.26, n = 47; βPix-NIKO, 2.02 ± 0.20, n = 52; *P* = 0.037).

Following this, the axons were identified based on Tau immunolabeling and their morphology was examined. We found that most of the longest axons were tau-positive axons at DIV4 and that the axons were shorter in the βPix-NIKO neurons than in the WT neurons (Fig 1E). In the βPix-NIKO neurons, the proportion of neurons with axons shorter than 100 μm was significantly higher and the proportion of those with axons longer than 200 μm was dramatically lower than in the WT neurons (Fig 1F). 36% of the WT neurons had axons longer than 200 μm, while only 12% of the βPix-NIKO neurons were in this group, consistent with the shorter average axon length in βPix-NIKO neurons than in WT neurons. Those results demonstrate that βPix neuronal isoforms are required for axonal outgrowth. The number of branching points in axons extending from the βPix-NIKO neurons was also lower than in the WT neurons (WT, 0.51 ± 0.06, n = 162; βPix-NIKO, 0.30 ± 0.04, n = 195; *P* = 0.003). However, there was no change in the number of axons between WT and βPix-NIKO mice neurons (WT, 1.34 ± 0.05, n = 120; βPix-NIKO, 1.24 ± 0.04, n = 158; *P* = 0.380), indicating that axon specification was not impaired by eliminating the expression of βPix neuronal isoforms. Collectively, these results demonstrate that βPix-NIKO neurons exhibit defects in neuronal morphogenesis during the neurite outgrowth and branching stages.

### Loss of neuronal βPix isoforms decreases tubulin acetylation in the longest neurite

**Fig 2:**
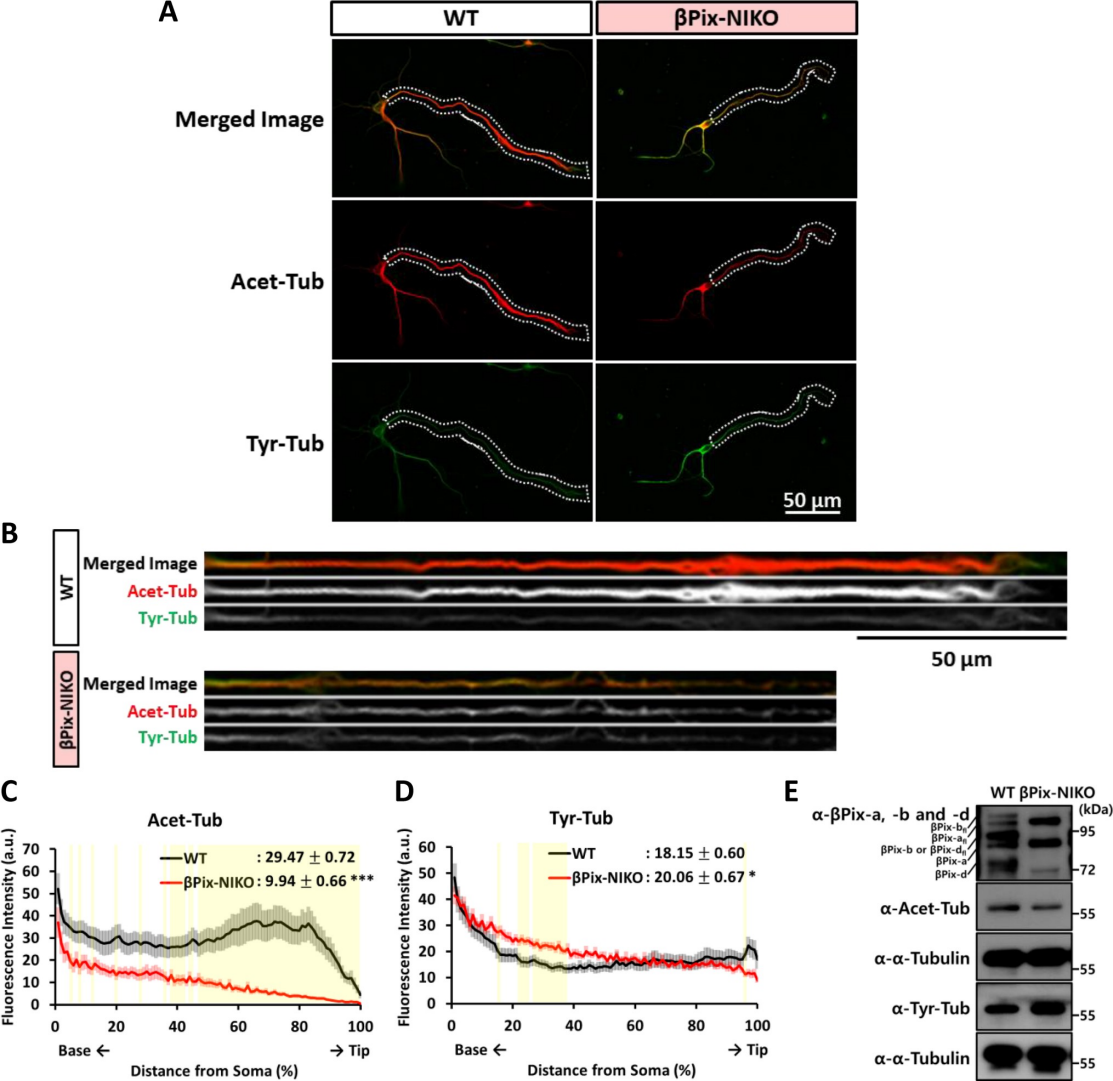
Tubulin acetylation is decreased in the longest neurite of the hippocampal neurons from βPix-NIKO mice. (A) Representative images of WT and βPix-NIKO neurons fixed at DIV4 and stained with acetylated α-tubulin (Acet-Tub) antibody (red) and tyrosinated α-tubulin (Tyr-Tub) antibody (green). The white dashed lines indicate the longest neurite of the neurons in each group and are straightened in Fig 2B. (B) The longest neurite of the WT and βPix-NIKO neurons shown in Fig 2A is straightened using ImageJ software. (C) The distribution graph shows that Acet-Tub levels are lower in the βPix-NIKO neurons throughout the length of the longest neurite compared with those in the WT neurons. The average Acet-Tub level (values in the top right corner) is 68% lower in the βPix-NIKO neurons compared with the WT neurons. (D) The distribution graph shows that Tyr-Tub levels slightly increase along the longest neurite extending from the βPix-NIKO neurons compared with that from the WT neurons. The average Tyr-Tub level (values in the top right corner) is 1.1-fold higher in the βPix-NIKO neurons compared with the WT neurons. n = 23–39 neurons per group from three independent cultures. (E) Representative blots showing Acet-Tub and Tyr-Tub levels in WT and βPix-NIKO neurons at DIV4. Compared with WT neurons, βPix-NIKO neurons show decreased levels in Acet-Tub and increased levels in Tyr-Tub. Data from at least three independent experiments. For the comparison of WT and βPix-NIKO neurons, * *P* < 0.05 and *** *P* < 0.001 for (C) and (D) by Student’s *t*-tests. The yellow boxes in (C) and (D) indicate *P* < 0.01 by Student’s *t*-tests.

Microtubule bundles constitute the core of developing neurites, with stability increasing as the neurons grow and become polarized [37]. During neurite outgrowth, one of multiple neurites extending from soma of hippocampal neurons elongates rapidly [36] and the longest neurite becomes the axon which is more enriched with long-lived stable microtubules compared to the dendrites [38]. Because neurons lacking the expression of βPix neuronal isoforms demonstrated marked impairment in the outgrowth of the longest neurite, which are particularly rich in stable microtubules, we hypothesized that microtubule stability would be disrupted in βPix-NIKO cultures. Acetylation of tubulin has been associated with microtubule stability and thus often used to label stable microtubules [39] while dynamic microtubules contain tyrosinated tubulin [40]. To examine post-translational modifications of tubulin in the longest neurite, we immunostained hippocampal neurons cultured from WT and βPix-NIKO mice at DIV4 with acetylated α-tubulin (Acet-Tub) antibody and tyrosinated α-tubulin (Tyr-Tub) antibody (Fig 2A). To quantify the levels of acetylated and tyrosinated tubulins along the length of the longest neurite, we straightened the microscopic image of the longest neurite and analyzed the distribution of the fluorescence intensity (Fig 2B). The mean level of Acet-Tub in the longest neurite in the βPix-NIKO neurons was significantly lower than that from WT neurons (Fig 2C), with the most dramatic difference observed close to the growing tip of the neurites (regions with *P* < 0.01 highlighted in yellow in Fig 2C). Conversely, the mean level of Tyr-Tub in the longest neurite was only slightly higher in the βPix-NIKO neurons, compared to the WT neurons (Fig 2D). Noticeable increases in the Tyr-Tub levels of the βPix-NIKO neurons were observed near the soma (regions with *P* < 0.01 highlighted in yellow in Fig 2D). Along with the immunofluorescence results, western blotting analysis showed that βPix-NIKO neurons display a decrease in the level of Ace-Tub and an increase in the level of Tyr-Tub (Fig 2E). Hence, our results demonstrate that βPix-NIKO neurons have defects in tubulin acetylation, indicating that βPix neuronal isoforms are required for the regulation of tubulin acetylation in developing hippocampal neurons.

### Recovery of microtubule stability by paclitaxel is sufficient to rescue impaired neurite morphology in βPix-NIKO neurons

**Fig 3:**
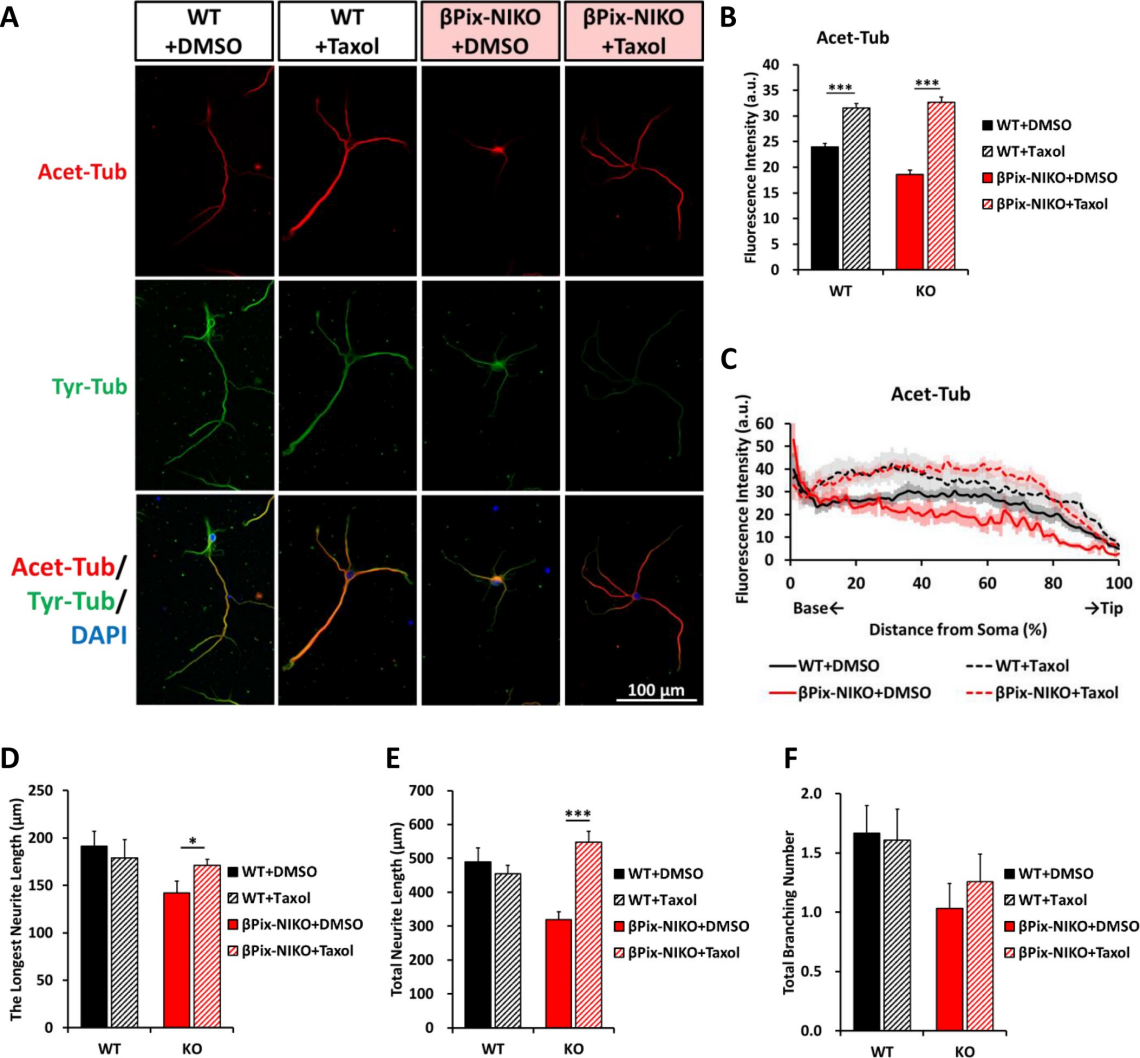
Impaired neuronal morphology in hippocampal neurons from neuronal βPix isoform KO mice is recovered by microtubule stabilization. (A) Representative images of WT and βPix-NIKO neurons treated with a vehicle (0.2% DMSO) or 4 nM paclitaxel (Taxol) at DIV1, fixed at DIV4, and stained with Acet-Tub antibody (red), Tyr-Tub antibody (green), and DAPI (blue). (B) Paclitaxel treatment elevates Acet-Tub levels in the longest neurite extending from the WT and βPix-NIKO neurons to comparable levels. (C) The distribution graph shows that Acet-Tub levels are increased by paclitaxel along the longest neurite in both the WT and βPix-NIKO cultures. (D) The reduction in the length of the longest neurite due to the loss of neuronal βPix isoforms is rescued by paclitaxel treatment. (E) The reduction in the total neurite length due to the loss of neuronal βPix isoforms is rescued by paclitaxel treatment. (F) Paclitaxel does not rescue the lower total branching number in the βPix-NIKO neurons. n = 27–33 neurons per group from three independent cultures. In (B), (D) and (E), * *P* < 0.05 and *** *P* < 0.001 by Student’s *t*-tests.

The precise regulation of microtubule stability is essential for neurite development [3]. Because the neurons cultured from βPix-NIKO mice exhibited the dysregulation of tubulin acetylation and defective neurite outgrowth, we hypothesized that the defects in the neuronal morphology observed in the βPix-NIKO neurons were caused by impaired microtubule stability. To test this hypothesis, we treated WT and βPix-NIKO hippocampal cultures with the microtubule-stabilizing reagent paclitaxel (Taxol) at DIV1 and incubated them for 72 h (Fig 3A). Following paclitaxel treatment, Acet-Tub levels increased in the longest neurite in both the WT and the βPix-NIKO neuron cultures (Fig 3B and 3C). Notably, the levels of Acet-Tub were comparable for the two genotypes, indicating that paclitaxel treatment was sufficient to rescue the loss of tubulin acetylation caused by the lack of neuronal βPix isoforms.

When we assessed the effect of paclitaxel on the neuronal morphology of WT neurons, we observed no change in the length of the longest neurite, total neurite length, or the number of branching points per neuron (Fig 3D–3F). However, neuronal morphology assessed using the same parameters was significantly affected by paclitaxel treatment in the βPix-NIKO neurons. The defects in the longest neurite length and total neurite length observed in the βPix-NIKO neurons were completely rescued by paclitaxel treatment (Fig 3D and 3E), demonstrating that reduced microtubule stability was the cause of the impaired neurite outgrowth observed in the βPix neuronal isoform-deficient neurons. The reduction in the total branching number observed in the βPix-NIKO neurons compared to the WT neurons was not remedied by paclitaxel treatment (Fig 3F). We suggest that microtubule stability in the WT neurons was sufficient for normal neurite outgrowth, and thus a further increase in microtubule stability due to paclitaxel does not affect neurite outgrowth. On the other hand, in the βPix-NIKO neurons, in which the lower tubulin acetylation leads to defects in neurite outgrowth, restoring tubulin acetylation with paclitaxel rescues impaired neurite outgrowth. Taken together, we suggest that the defects in neuronal morphology observed in the βPix-NIKO neurons result from disrupted tubulin acetylation and microtubule stability and that βPix neuronal isoforms are essential for the regulation of tubulin acetylation.

### βPix-d is the primary neuronal isoform required for tubulin acetylation

**Fig 4:**
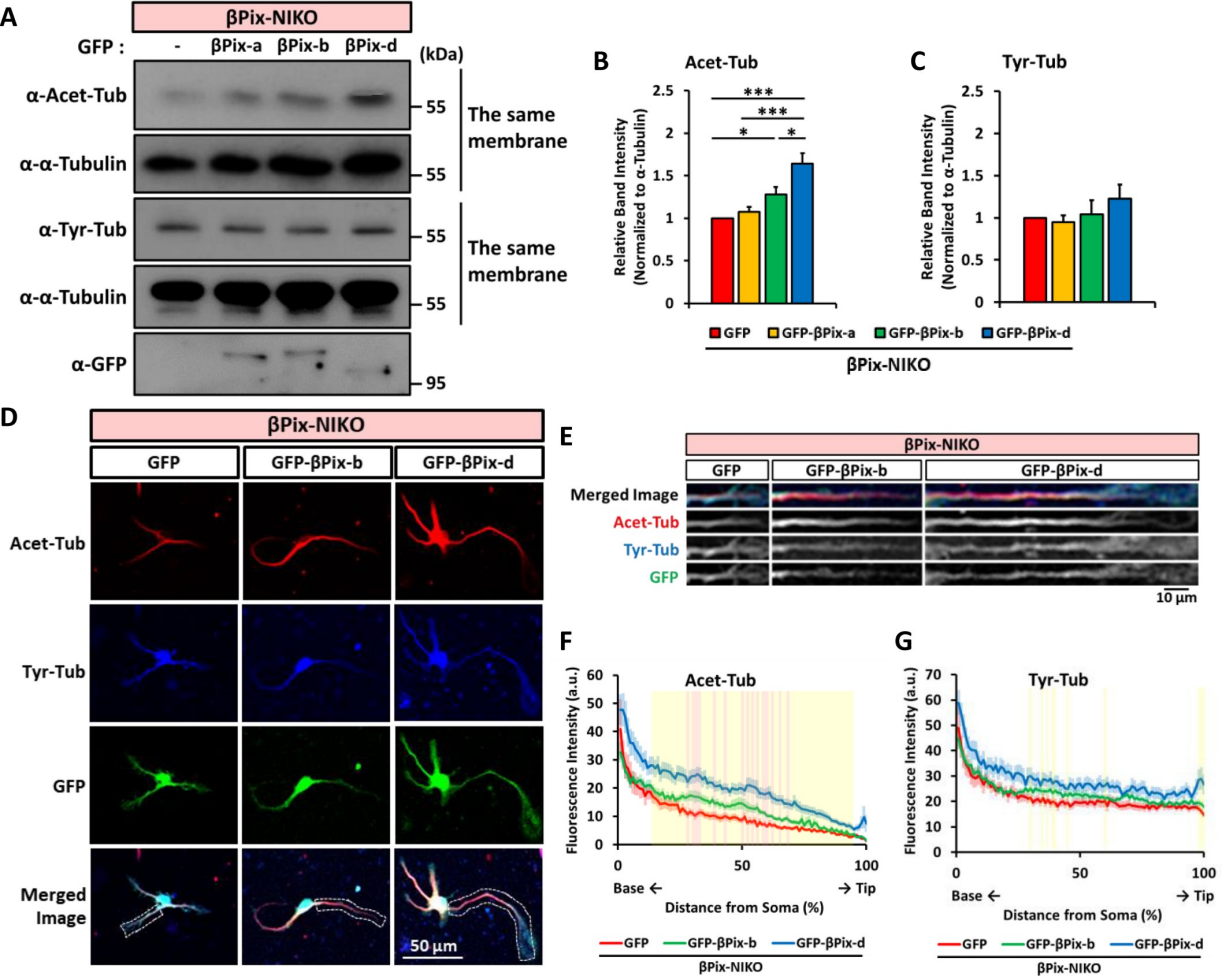
βPix-b and βPix-d regulate tubulin acetylation. (A) Representative blots showing Acet-Tub and Tyr-Tub levels in βPix-NIKO neurons transfected with GFP, GFP-βPix-a, GFP-βPix-b, or GFP-βPix-d at DIV3 and lysed at DIV4. (B) Quantification of relative Acet-Tub expression levels for the results shown in (A) as normalized to α-tubulin expression. GFP-βPix-b or GFP-βPix-d induces a significant increase in the level of Acet-Tub, with GFP-βPix-d having a greater effect than GFP-βPix-b. (C) Quantification of relative Tyr-Tub expression levels for the results shown in (A) as normalized to α-tubulin expression. There is no significant difference in Tyr-Tub levels for the expression of GFP-βPix-a, GFP-βPix-b, or GFP-βPix-d in the βPix-NIKO neurons. (D) Representative images of βPix-NIKO neurons transfected with GFP, GFP-βPix-b, or GFP-βPix-d at DIV3, fixed at DIV4, and stained with Acet-Tub antibody (red) and Tyr-Tub antibody (blue). The white dashed lines indicate the longest neurite of the neuron in each group, which is straightened in Fig 4E. (E) The longest neurite of the βPix-NIKO neurons transfected with GFP, GFP-βPix-b, or GFP-βPix-d from Fig 4D is straightened using ImageJ software. (F) The Acet-Tub levels along the longest neurite are increased by GFP-βPix-b or GFP-βPix-d expression in the βPix-NIKO neurons compared with the GFP-expressing control. (G) There is little significant change in Tyr-Tub levels along the longest neurite following the expression of GFP-βPix-b or GFP-βPix-d in the βPix-NIKO neurons. Five independent cultures for (A)–(C) and n = 61–82 neurons per group from three independent cultures for (D)–(G). For the comparison among hippocampal neurons from neuronal βPix isoform KO mice transfected with GFP, GFP-βPix-b, and GFP-βPix-d, * *P* < 0.05 and *** *P* < 0.001 for (B) by one-way ANOVA with post-hoc Tukey’s test. Pink boxes in (F) denote *P* < 0.01 for the comparison of GFP and GFP-βPix-b and yellow boxes in (F) and (G) denote *P* < 0.01 for the comparison of GFP and GFP-βPix-d by Student’s *t*-tests.

Next, we set out to identify which of the neuron-specific βPix isoforms βPix-b and βPix-d is responsible for the regulation of tubulin acetylation during neuronal morphogenesis. To restore the expression of each isoform in neurons lacking βPix neuronal isoforms, we transfected hippocampal neurons from βPix-NIKO mice at DIV3 with GFP, GFP-βPix-a, GFP-βPix-b, or GFP-βPix-d (Fig 4A). Quantification of Acet-Tub levels using Western blotting revealed that expressing either GFP-βPix-b or GFP-βPix-d resulted in a significant increase in the levels of Acet-Tub at DIV4 compared to the expression of GFP alone (Fig 4A and 4B). In contrast, the expression of the ubiquitous βPix-a did not alter Acet-Tub levels (Fig 4A and 4B). Notably, the increase by GFP-βPix-d expression (1.64 ± 0.12) was significantly greater than that by GFP-βPix-b expression (1.28 ± 0.09) (Fig 4B). In contrast, Tyr-Tub levels were not significantly altered by the transfection of any βPix isoform compared with the GFP control (Fig 4C), confirming that βPix neuronal isoforms regulate tubulin modifications mainly by increasing the level of tubulin acetylation rather than affecting tubulin tyrosination.

Additionally, we investigated the regulation of post-translational modification in tubulins using immunofluorescence in order to rule out the effect of non-neuronal cells in the culture. βPix-NIKO neurons were transfected at DIV3 with GFP, GFP-βPix-b, or GFP-βPix-d and immunostained with Acet-Tub antibody and Tyr-Tub antibody at DIV4 (Fig 4D). We straightened the microscopic image of the longest neurite and analyzed the distribution of the fluorescence intensity (Fig 4E). Consistent with the results from Western blotting analysis, the expression of GFP-βPix-d resulted in a significantly higher increase in Acet-Tub levels of the longest neurite compared to the expression of GFP-βPix-b (significant regions with *P* < 0.01 highlighted in pink for GFP-βPix-b vs. GFP and yellow for GFP-βPix-d vs. GFP in Fig 4F). Additionally, we also identified that the effect of overexpressing GFP-βPix-b or GFP-βPix-d in βPix-NIKO neurons was sufficient to increase the Acet-Tub levels higher than those in WT neurons expressing GFP (S1 Fig). Tyr-Tub was increased by GFP-βPix-d expression in very limited regions (Fig 4G). Overall, these results indicate that neuronal βPix isoforms enhance tubulin acetylation, with βPix-d exerting a much stronger effect on tubulin acetylation than the βPix-b isoform.

A previous study by our group showed that βPix-b is localized in dendritic spines that are particularly rich in F-actin [32]. However, the subcellular localization of βPix-d in neurons has not been identified. To examine the location of βPix-d in relation to the cytoskeleton, we labeled endogenous βPix-d in cultured WT neurons with an antibody against 11 βPix-d-specific a.a. (S2 Fig) and counterstained the neurons with phalloidin and β3-tubulin antibody, which labeled F-actin and neuronal microtubules, respectively (S3A Fig). We analyzed the straightened neurite images to quantify the fluorescence intensity along the longest neurite extending from the neurons (S3B Fig). F-actin is known to be present in high levels in motile growth cones and branch initiation points in neurite shafts [41], while microtubules are commonly found throughout neurites. We found that F-actin levels were consistently high in the neurite tip and at particular points in the shaft, whereas β3-tubulin was distributed throughout the neurite (S3C Fig, red and blue lines, respectively). Interestingly, the distribution of βPix-d was comparable to that of β3-tubulin but differed from that of F-actin (S3C Fig). The disparity was apparent in the ratio of βPix-d intensity to β3-tubulin intensity (βPix-d/β3-tubulin, the pink line in S3C Fig) and in the ratio of βPix-d intensity to F-actin intensity (βPix-d/F-actin, the yellow line in S3C Fig). The βPix-d/β3-tubulin ratio remained roughly unchanged along the length of neurites, while the βPix-d/F-actin ratio fluctuated along the length of neurites, suggesting that the βPix-d was localized closely to the microtubules. This localization pattern differs from the previously reported localization of βPix-b in F-actin-rich compartments [32] and supports distinct roles of the neuronal βPix isoforms during neuronal development.

### βPix-d is required for the phosphorylation of Stathmin1 at Ser16 and neurite outgrowth

**Fig 5:**
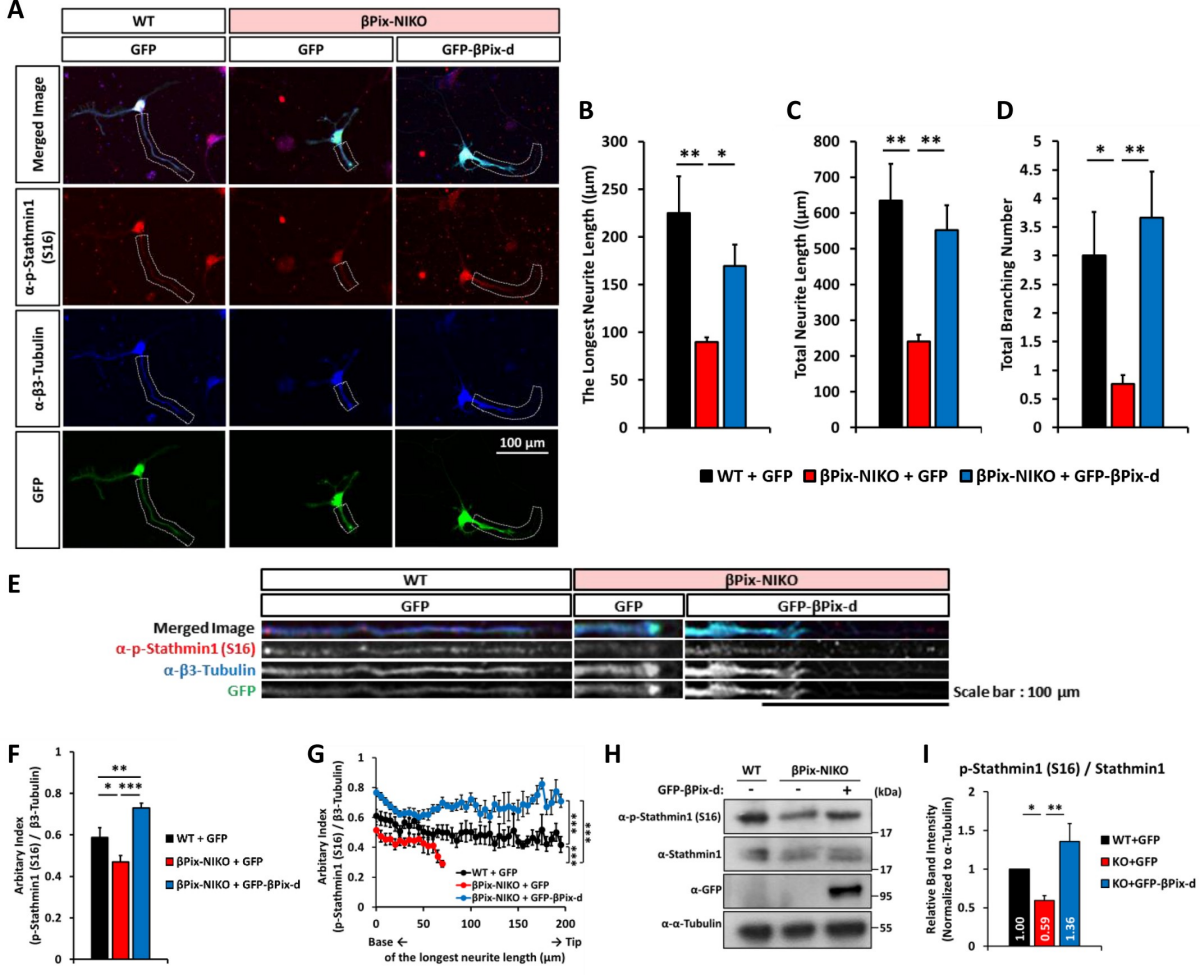
βPix-d regulates neurite outgrowth and the phosphorylation of Stathmin1 at Ser16. (A) Representative images of WT neurons transfected with GFP and βPix-NIKO neurons transfected with GFP or GFP-βPix-d at DIV3, fixed at DIV4, and stained with p-Stathmin1 (S16) (red) and β3-tubulin antibodies (blue). (B) The length of the longest neurite decreases by 60% in the βPix-NIKO neurons expressing GFP (NIKO+GFP) compared with the WT neurons transfected with GFP (WT+GFP). The expression of GFP-βPix-d in the βPix-NIKO neurons rescues the defects in neurite length. (C) Total neurite length decreases by 62% in the NIKO+GFP neurons compared with the WT+GFP neurons. The expression of GFP-βPix-d in the βPix-NIKO neurons rescues defects in neurite length. (D) Total branching number decreases by 73% in the NIKO+GFP neurons compared with the WT+GFP neurons. The expression of GFP-βPix-d in the βPix-NIKO neurons rescues the defect in branch numbers. (E) The longest neurite shown in Fig 5A are straightened using ImageJ software. (F) In the longest neurite, the phosphorylation levels of Stathmin1 at Ser16, which are normalized to β3-tubulin, decreases by 20% in the NIKO+GFP neurons compared with the WT+GFP neurons. The expression of GFP-βPix-d in the βPix-NIKO neurons rescues the phosphorylation levels of Stathmin1 at Ser16. (G) In the longest neurite, the phosphorylation of Stathmin1 at Ser16 is significantly lower in the NIKO+GFP neurons compared with the WT+GFP neurons and is recovered by the expression of GFP-βPix-d in the βPix-NIKO neurons. (H) Representative blots for the phosphorylation level of Stathmin1 at Ser16 in WT neurons transfected with GFP and βPix-NIKO neurons transfected with GFP or GFP-βPix-d at DIV3 and lysed at DIV4. (I) Quantification of relative expression levels for the results shown in (H) as normalized to α-tubulin expression. Compared with the WT+GFP neurons, the NIKO+GFP neurons show a 41% decrease in the ratio of p-Stathmin1 at Ser16 to total Stathmin1, and this ratio is recovered by the expression of GFP-βPix-d in the βPix-NIKO neurons. n = 30–35 neurons per group from three independent cultures for (A)–(G) and six independent cultures for (H) and (I). In (B)–(D), (F), (G), and (I), * *P* < 0.05, ** *P* < 0.01, and *** *P* < 0.001 by one-way ANOVA followed by post-hoc Tukey’s test.

We found that βPix-d promotes tubulin acetylation and is localized with the microtubules in neurites. Therefore, we tested whether the expression of βPix-d is sufficient for the regulation of neurite outgrowth by βPix. We transfected GFP-βPix-d to hippocampal neurons cultured from βPix-NIKO mice at DIV3 and examined whether the expression of βPix-d rescues the reduced neurite length and branching number in βPix-NIKO cultures at DIV4 (Fig 5A). As shown in Fig 1, the length of the longest neurite, total neurite length, and total branching number were lower in the absence of βPix neuronal isoforms compared with the WT neurons. These defects disappeared with the sole expression of the βPix-d isoform in the βPix-NIKO neurons (Fig 5B–5D). These results strongly indicate that the βPix-d isoform regulates neurite morphogenesis.

Next, we set out to identify the mechanisms by which βPix-d regulates tubulin acetylation and neurite morphogenesis. We focused on Stathmin1, an essential regulator of microtubule stability associated with neuronal differentiation and plasticity [42] because it is well-known that Stathmin1 activity is regulated by PAK kinase, which is directly associated with and regulated by βPix [18]. Of the four phosphorylation sites in Stathmin1, phosphorylation at Ser16 is catalyzed by PAK [19] and supports microtubule stability by inhibiting the sequestering of tubulin dimers [10]. Using immunostaining analysis (Fig 5A), we found that the level of phosphorylation at Ser16 of Stathmin1 in the longest neurite was lower in the βPix-NIKO neurons than in the WT neurons (Fig 5A and 5E–5G). The expression of βPix-d in the βPix-NIKO neurons rescued the mean level and distribution of p-Stathmin1 (Ser16), suggesting that βPix-d is the necessary and sufficient βPix isoform for the phosphorylation of Stathimin1 at Ser16 in developing neurites (Fig 5F and 5G). In addition, in Western blotting analysis, we consistently observed that the phosphorylation levels of Stathmin1 at Ser16 is dependent on βPix-d (Fig 5H). The βPix-NIKO neurons exhibited a 41% decrease in the phosphorylation levels of Stathmin1 at Ser16 compared with the WT neurons, which was rescued by the expression of βPix-d (Fig 5I). Taken together, we found that βPix-d is required for neurite outgrowth and the phosphorylation of Stathmin1 at Ser16.

The present study found that both βPix-b and βPix-d support tubulin acetylation (Fig 4). Therefore, we also investigated the role of βPix-b in neurite outgrowth and the phosphorylation of Stathmin1. First, we transfected hippocampal neurons from βPix-NIKO mice at DIV3 with GFP, GFP-βPix-b, or GFP-βPix-d and analyzed the neurite length and p-Stathmin1 levels at DIV4 (S4A Fig). The expression of GFP-βPix-b did not significantly change the longest neurite length or branching numbers, but significantly increased total neurite length (S4B–S4D Fig). Next, to investigate the recovery of p-Stathmin1 (Ser16) levels by βPix-b, we analyzed the immunofluorescence intensity of p-Stathmin1 (Ser16) along the longest neurite extending from the βPix-NIKO neurons expressing GFP-tagged neuronal βPix isoforms (S4A and S4E Fig). We found that the expression of either βPix-b or βPix-d recovered Stathmin1 phosphorylation levels at Ser16 (S4F and S4G Fig). Consistent with the results for neurite outgrowth, the expression of the βPix-d isoform increased p-Stathmin1 (Ser16) levels to a significantly greater extent than did βPix-b expression (S4F and S4G Fig). Collectively, these results suggest that both βPix-b and βPix-d can control the phosphorylation of Stathmin1 (Ser16) and neurite outgrowth, with βPix-d playing a dominant role.

### Regulation of stathmin1 (Ser16) phosphorylation and neurite outgrowth by βPix-d is PAK-dependent

**Fig 6:**
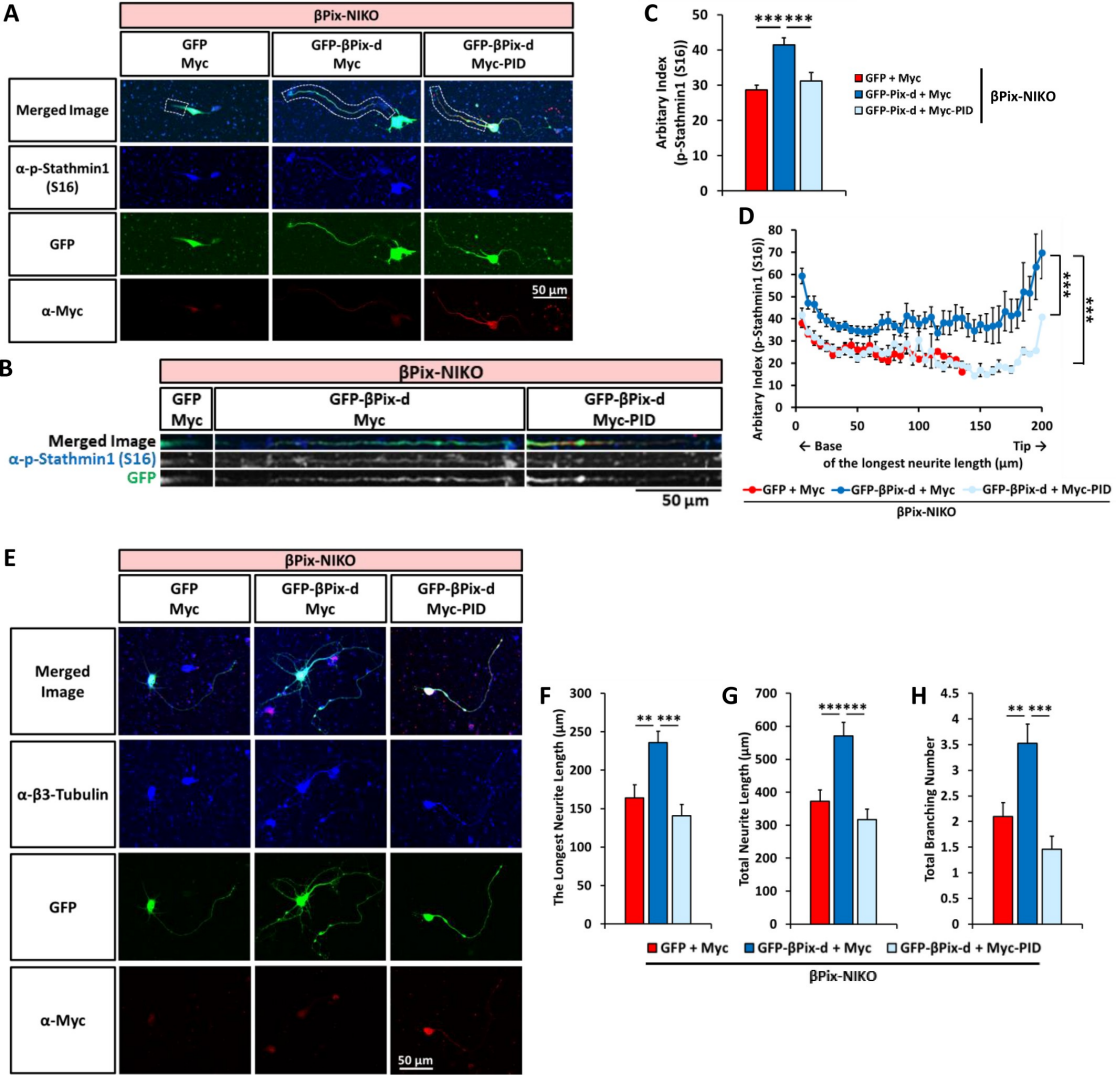
PAK is required for βPix-d-induced phosphorylation of Stathmin1 at Ser16 and neurite outgrowth. (A) Representative images of βPix-NIKO neurons transfected with GFP and Myc, GFP-βPix-d and Myc, or GFP-βPix-d and Myc-PID at DIV3, fixed at DIV4, and stained with p-Stathmin1 (S16) (blue) and Myc antibodies (red). (B) The longest neurites shown in Fig 6A are straightened using ImageJ software. (C) The expression of GFP-βPix-d elevates the average phosphorylation levels of Stathmin1 at Ser16 in the longest neurite extending from the βPix-NIKO neurons, but the co-expression of Myc-PID with GFP-βPix-d removes the increase. (D) In the longest neurite extending from the βPix-NIKO neurons, expression of GFP-βPix-d recovers the phosphorylation levels of Stathmin1 at Ser16 but the co-expression of GFP-βPix-d and Myc-PID does not. (E) Representative images of βPix-NIKO neurons transfected with GFP and Myc, GFP-βPix-d and Myc, or GFP-βPix-d and Myc-PID at DIV3, fixed at DIV4, and stained with β3-Tubulin (blue) and Myc antibodies (red). (F) In the βPix-NIKO neurons, an increase in the longest neurite length by GFP-βPix-d is not observed when Myc-PID is co-expressed with GFP-βPix-d. (G) In the βPix-NIKO neurons, an increase in total neurite length by GFP-βPix-d is not observed when Myc-PID is co-expressed with GFP-βPix-d. (H) In the βPix-NIKO neurons, an increase in the total branching number by GFP-βPix-d is not observed when Myc-PID is co-expressed with GFP-βPix-d. n = 45–56 neurons per group from 3 independent cultures for (A)–(D) and n = 67–84 neurons per group from 3 independent cultures for (E)–(H). In (C), (D), and (F)–(H), ** *P* < 0.01 and *** *P* < 0.001 by one-way ANOVA followed by post-hoc Tukey’s test.

PAK is a well-known βPix-interacting protein and can phosphorylate Ser16 of Stathmin1 [19]. To determine whether the βPix-d-dependent regulation of Stathmin1 phosphorylation at Ser16 is mediated by PAK, we utilized the expression of a PID construct, which leads to the inactivation of PAK activity. We co-transfected hippocampal neurons cultured from βPix-NIKO mice with GFP-βPix-d and myc-PID to examine the effect of the inhibition of PAK activity (Fig 6A). Expressing GFP-βPix-d alone in the βPix-NIKO neurons resulted in an increase in the Stathmin1 phosphorylation levels at Ser16. Strikingly, the increase in mean Stathmin1 phosphorylation levels was completely removed with the co-expression of myc-PID (Fig 6B and 6C). Plotting p-Stathmin1 (Ser16) intensity along the longest neurite led to the same conclusion that the induction of Stathmin1 phosphorylation at Ser16 through GFP-βPix-d requires PAK activity (Fig 6D). Because PAK is a kinase that is known to phosphorylate Stathmin1, these results strongly suggest that βPix-d induces the phosphorylation of Stathmin1 at Ser16 by promoting direct phosphorylation via PAK. Interestingly, Myc-PID expression in βPix-NIKO neurons leads to marked decreases in the longest neurite length, total neurite length and total branching number (S5A–S5D Fig), but merely a slight decrease in the phosphorylation of Stathmin1 at Ser16, compared with the βPix-NIKO neurons transfected with the control vectors (S5E–S5H Fig). These data indicate that PAK activity phosphorylating Stathmin1 is specifically induced by βPix-d, while the role of PAK in neuronal morphogenesis can be also regulated by βPix-d-independent mechanisms.

**Fig 7:**
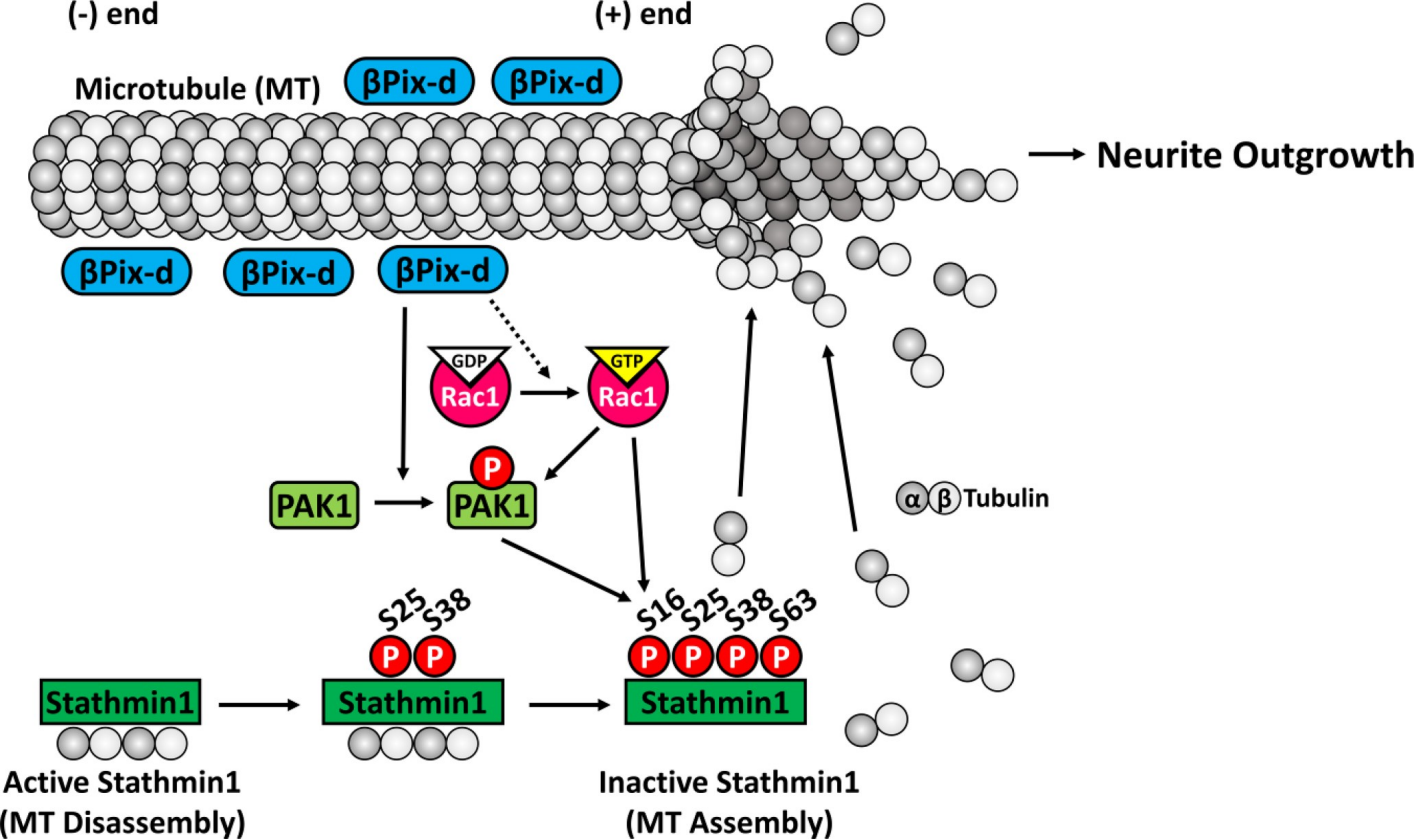
Model illustrating the regulation of microtubule stabilization and neurite outgrowth by βPix-d. βPix-d localized around microtubules activates PAK and, in turn, the active PAK phosphorylates Stathmin1 at Ser16. Phosphorylated Stathmin1 in an inactive state releases α- and β-tubulin heterodimers for microtubule polymerization and inhibits microtubule catastrophe in neurites. In summary, βPix-d promotes microtubule stabilization and neurite outgrowth.

Finally, we examined whether PAK is involved in the promotion of neurite outgrowth by βPix-d by testing whether the recovery of defective neurite morphology in the βPix-NIKO neurons by rescuing βPix-d is dependent on PAK activity. We analyzed the morphology of neurons co-expressing GFP-βPix-d and myc-PID using immunostaining with β3-tubulin antibody at DIV4 (Fig 6E). The length of the longest neurite, total neurite length, and total branching numbers were restored following the expression of βPix-d, but the co-expression of PID prevented the recovery of neurite extension (Fig 6F–6H). Therefore, our results demonstrate that the role of the βPix neuronal isoform in promoting neurite outgrowth and the phosphorylation of Stathmin1 is mediated by PAK activity. In conclusion, our results show that βPix neuronal isoforms, mainly βPix-d, promote tubulin acetylation and neurite outgrowth via the PAK-dependent phosphorylation of Stathmin1 at Ser16 (Fig 7).

## Discussion

The present study reveals a novel signaling pathway in which βPix neuronal isoforms promote neurite outgrowth in developing neurons. βPix is known to be essential for the activation of the small GTPases Rac1 and Cdc42, which in turn activate PAK kinase [20, 21]. Our results support a model in which the βPix neuronal isoforms promote Stathmin1 phosphorylation by regulating PAK activity, which is required for microtubule stabilization during neurite development [19]. Through alternative splicing, the *ARHGEF7* gene produces a ubiquitously expressed βPix-a isoform and the neuronally expressed βPix isoforms βPix-b and βPix-d, which have distinct domain compositions [29]. The expression of βPix-b and βPix-d is highly specific to neurons, and they are present in high levels throughout the developmental stages [32], suggesting that they have an important role in neuronal development. Our recent study revealed a novel mechanism by which βPix-b promotes dendritic spine and synapse formation [32]. However, to date, the specific role of each neuronal isoform has remained unclear. Importantly, we revealed the neuronal role of the βPix-d isoform for the first time, finding that βPix-d is required for neurite morphogenesis in developing neurons. We observed that both βPix neuronal isoforms can regulate Stathmin1 phosphorylation at Ser16, tubulin modification and microtubule stabilization, and neurite outgrowth. However, the defects in neurite morphology and Stathmin1 phosphorylation were more strongly affected by the expression of βPix-d than by βPix-b in the βPix-NIKO hippocampal cultures. βPix-d was closely localized to microtubules and is thus likely to efficiently activate PAK near microtubules, thereby acting as a major βPix isoform regulating Stathmin1 phosphorylation. Stathmin1 phosphorylation is associated with microtubule polymerization and stabilization by reducing the sequestration of tubulin heterodimers and neurite extension [10, 15].

Several studies have suggested that βPix is involved in neurite outgrowth. αPix, encoded by *ARHGEF6*, has an 80% sequence homology with βPix protein and has been identified as a specific regulator of axonal and dendritic branching in hippocampal neurons [43]. The βPix/Ras/ERK/PAK2 pathway is also involved in fibroblast growth factor-induced neurite outgrowth in PC12 cells [44], and βPix promotes axon formation as an upstream activator of TC10, which is closely related to Cdc42 [25]. Although previous studies have revealed the role of βPix in the extension of neuronal processes, those studies did not identify isoform-specific functions but mainly focused on the ubiquitous βPix-a isoform [26], in part due to the difficulty of isoform-specific genetic analysis. Based on the βPix domain structure, in which the neuronal isoforms share the INS domain encoded by exon 19, we generated a βPix-NIKO mouse line in which the expression of the neuronal isoforms is specifically blocked. Because the constitutive KO of all βPix isoforms results in embryonic lethality [45], this isoform-specific KO model allowed us to study the roles of neuronal βPix isoforms. By expressing each neuronal isoform in a βPix-NIKO background, we were able to determine their distinctive roles. The role of βPix-b in dendritic spine morphogenesis was reported in a recent study utilizing the βPix-NIKO model [32], and the present study addressed the role of βPix-d in neurite outgrowth.

We suggest that βPix-d may be directly associated with microtubules. Murine Lfc, its human homologue GEF-H1, and p190RhoGEF are members of the Dbl family GEFs, like βPix, and they bind to microtubules [46–48], suggesting a potential interaction between βPix and microtubules. Lfc binds to microtubules through the PH domain [48], supporting the possibility that βPix-d might interact with microtubules via the PH domain. However, the PH domain is shared by all βPix isoforms, which raises the question of the mechanisms underlying the specific localization of the βPix-d isoform with microtubules. Unlike βPix-a and βPix-b, βPix-d does not contain a PDZ-binding motif or LZ domain at the C terminus. This lack of the two functional sequences might explain the isoform-specific localization. The PDZ-binding sequence of βPix interacts with the PDZ domain in Shank [24] and Scribble [49]. Shank proteins are multidomain scaffold proteins of the postsynaptic density that connect synaptic proteins to the actin cytoskeleton [50] and Scribble is a peripheral membrane scaffold protein involved in cell polarity and neuronal morphogenesis [51]. The LZ domain mediates the localization of βPix in the cell periphery and is also responsible for βPix dimerization [21]. We speculate that βPix-a and βPix-b are mainly dimerized or interact with Shank or Scribble in the dendritic spine, which may hinder their binding to microtubules. In addition, βPix-d contains 11 a.a. at the C terminal that are specific to βPix-d. Hence, future research into the role of the 11 a.a. region will increase the understanding of the localization and function of βPix-d.

Of the Rac/Cdc42 GEFs, DOCK7 has a functional similarity to βPix-d, with Watabe-Uchida and colleagues reporting that DOCK7 regulates Rac activity and inactivates Stathmin1 [16]. Notably, they demonstrate that DOCK7 plays a role in neuronal polarization and axon formation during the early stages of neuronal development. In contrast, we found no significant difference in neurite and axon numbers between WT and βPix-NIKO mice, indicating that βPix neuronal isoforms are not required for neurite formation and neuronal polarization. Instead, our results show that expression of βPix-d in βPix-NIKO neurons recovers neurite length and branching number in neurites, suggesting that βPix-d plays a role in the outgrowth of neurites, including both axons and dendrites. In accordance with our findings, Stathmin1, the effector of the βPix pathway, is highly expressed in the nervous system during brain development [42] and regulates the development of axons [16] and dendrites [17], with its activity controlled by phosphorylation at Ser16. Therefore, our research suggests that βPix and Stathmin1 work together to regulate neurite development at the neurite extension stage.

Various studies have reported that changes in dendrite morphology or defects in neuronal development contribute to several neuropsychiatric and neurodevelopmental disorders [52]. In addition, microtubule stability is involved in not only neuronal morphogenesis [53] but also normal cognitive function [54]. Knocking out Stathmin1 in mice results in mostly mild phenotypes in neuronal development and structures [42, 55], suggesting that the misregulation of Stathmin1 phosphorylation in βPix-NIKO cultures might have only partially contributed to the impaired neurite outgrowth. Rather, Stathmin1 has been linked to fear, cognition, and aging in rodent and human studies [56–60], and Stathmin1-dependent changes in microtubule stability are involved in synaptic function and memory formation [54]. To date, there has been no research reported on memory or behavior dysfunction resulting from the removal of βPix isoforms, so it would be interesting to examine βPix-NIKO mice for these phenotypes. It is worth noting that our βPix-NIKO mice were viable, in contrast to the complete KO of all βPix isoforms, which is embryonically lethal. Thus, our mouse line has the potential to be a powerful model for testing the effects of neuronal isoforms on memory formation and disease-related behavior.

The regulation of microtubule stability might also be a useful approach for the treatment of neurodegenerative disorders and central nervous system injury. Microtubule stability is required for the maintenance of neuronal structure and function, as indicated by the microtubule stability defects observed in a number of neurodegenerative diseases. Reduced microtubule stability has been observed in Alzheimer’s disease, Parkinson’s disease, and amyotrophic lateral sclerosis, whereas hyperstable microtubules have been observed in hereditary spastic paraplegia [61–64]. The pharmacological induction of microtubule stability has been shown to promote axonal regeneration after spinal cord injury [65]. Thus, the βPix-dependent regulation of microtubule stabilization and neurite outgrowth established in the present study may offer a basis for the development of a method to recover the impaired neural function associated with degenerative disorders and injuries. Further studies are essential to determine the specific involvement of neuronal βPix isoforms in these diseases and regeneration processes.

In conclusion, we have outlined a novel pathway that regulates neurite outgrowth and tubulin acetylation, and our findings identify the specific role of βPix neuronal isoforms, with a particular focus on the βPix-d isoform. βPix-b and βPix-d are required for neurite development and the regulation of microtubule stability through the phosphorylation of Stathmin1 at Ser16. βPix-d is localized with microtubules and our results link βPix-d to the local inactivation of the microtubule-destabilizing protein Stathmin1. Future research, including the real-time imaging of microtubules and biochemical examination of association between microtubules and βPix-d, will help to specify the role of βPix-d in the regulation of microtubule dynamics.

## Supporting information

S1 Fig βPix-b and βPix-d are important for tubulin acetylation. (A) Representative images of WT neurons transfected with GFP and βPix-NIKO neurons transfected with GFP, GFP-βPix-b, or GFP-βPix-d at DIV3, fixed at DIV4, and stained with Acet-Tub antibody (red) and Tyr-Tub antibody (blue). The white dashed lines indicate the longest neurite of the neuron in each group, which is straightened in S1B Fig. (B) The longest neurite of the WT neurons transfected with GFP and βPix-NIKO neurons transfected with GFP, GFP-βPix-b, or GFP-βPix-d from S1A Fig is straightened using ImageJ software. (C) The longest neurite extending from βPix-NIKO neurons has lower mean level of Acet-Tub than that from WT neurons. In βPix-NIKO neurons, expression of βPix-b or βPix-d recovers the decreased mean level of tubulin acetylation and the recovery level of tubulin acetylation was higher than WT neurons. (D) The distribution graph showed that reduced Acet-Tub level in βPix-NIKO neurons was observed, compared with that in WT neuron. The decrease in Acet-Tub was rescued by βPix-b or βPix-d expression in βPix-NIKO neurons. The rescued level was higher than the tubulin acetylation that WT neurons have. n = 18–30 neurons for each group. In (C), *** *P* < 0.001 by one-way ANOVA followed by post-hoc Tukey’s test.
(TIF)

S2 Fig In immunocytochemistry, anti-11 a.a. antibody specifically detects βPix-d. After expression of GFP-βPix-b or GFP-βPix-d in βPix-KO MEFs (35), those MEFs were stained with anti-11 a.a. antibody. By anti-11 a.a. antibody, βPix-b was not detected, but βPix-d was detected. Data from three independent cultures.
(TIF)

S3 Fig βPix-d is co-localized with microtubules. (A) Representative images of WT neurons fixed at DIV4 and stained with anti-11 a.a. antibody (green), anti-β3-tubulin antibody(blue), and rhodamine-conjugated phalloidin (red). The white dashed lines indicate the longest neurite of the WT neurons, which is straightened in (B). (B) The axon extending from the WT neurons shown in (A) is straightened using ImageJ software. (C) βPix-d is co-localized with microtubules and not with F-actin. n = 21 neurons from three independent cultures.
(TIF)

S4 Fig βPix-b and βPix-d regulate neurite outgrowth and the phosphorylation of Stathmin1 at Ser16. (A) Representative images of βPix-NIKO neurons transfected with GFP, GFP-βPix-b, or GFP-βPix-d at DIV3, fixed at DIV4, and stained with p-Stathmin1 (S16) (red) and β3-tubulin (blue) antibodies. (B) The longest neurite length increases with the expression of GFP-βPix-b or GFP-βPix-d in the βPix-NIKO neurons compared with the GFP-expressing control. (C) Total neurite length is recovered by the expression of GFP-βPix-b or GFP-βPix-d in the βPix-NIKO neurons compared with the GFP-expressing control. (D) Total branching number is recovered by the expression of GFP-βPix-b or GFP-βPix-d in the βPix-NIKO neurons compared with the GFP-expressing control. (E) The longest neurites shown in (A) are straightened using ImageJ software. (F) Phosphorylation levels of Stathmin1 at Ser16 in the longest neurite are recovered by expressing GFP-βPix-b or GFP-βPix-d in βPix-NIKO neurons compared with the GFP-expressing control. The phosphorylated Stathmin1 levels are normalized to β3-tubulin. (G) The phosphorylation of Stathmin1 at Ser16 is recovered by expressing GFP-βPix-b or GFP-βPix-d compared with the GFP-expressing control along the longest neurite extending from the βPix-NIKO neurons. n = 61–82 neurons per group from three independent cultures. In (B)–(D), (G) and (H), * *P* < 0.01 and *** *P* < 0.001 by one-way ANOVA followed by post-hoc Tukey’s test.
(TIF)

S5 Fig Inhibition of PAK activity in βPix-NIKO neurons results in defective neurite outgrowth and largely maintains the phosphorylation of Stathmin1 at Ser16. (A) Representative images of βPix-NIKO neurons transfected with GFP and Myc or GFP and Myc-PID at DIV3, fixed at DIV4, and stained with β3-Tubulin (blue) and Myc antibodies (red). (B–D) In the βPix-NIKO neurons, expression of GFP and Myc-PID decreased the longest neurite length (B), total neurite length (C), and total branching number (D), compared with expression of GFP and Myc. (E) Representative images of βPix-NIKO neurons transfected with GFP and Myc or GFP and Myc-PID at DIV3, fixed at DIV4, and stained with p-Stathmin1 (S16) (blue) and Myc antibodies (red). (F) The longest neurites shown in S5E Fig are straightened using ImageJ software. (G) In the longest neurite, the mean level of Stathmin1 phosphorylation at Ser16 was slightly decreased in βPix-NIKO neurons transfected with GFP and Myc-PID, compared with GFP and Myc. (H) Along the longest neurite, a slightly decrease of phosphorylated Stathmin1 level at Ser16 was observed with expression of GFP and Myc-PID, compared with GFP and Myc in βPix-NIKO neurons. n = 35–89 neurons per group for (B)–(D) and n = 52–67 neurons per group for (G) and (H). In (B)–(D) and (G), *** *P* < 0.001 by one-way ANOVA followed by post-hoc Tukey’s test.
(TIF)

S6 Fig Original uncropped images underlying all blot results. (PDF)
