# Correction: βPix-d promotes tubulin acetylation and neurite outgrowth through a PAK/Stathmin1 signaling pathway

**DOI:** 10.1371/journal.pone.0233327

**Published:** 2020-05-13

**Authors:** Younghee Kwon, Ye Won Jeon, Minjae Kwon, Yongcheol Cho, Dongeun Park, Jung Eun Shin

The following information is missing from the Funding statement: Y.C. was supported by the National Research Foundation of Korea (NRF) grant funded by the Korea government (MSIT) (NRF-2019R1A2C1005380).
