# Supplementary figures and images for: βPix-d promotes tubulin acetylation and neurite outgrowth through a PAK/Stathmin1 signaling pathway
Source: PLoS One. 2020 Apr 6;15(4):e0230814. doi: 10.1371/journal.pone.0230814 (PMC7135283; doi:10.1371/journal.pone.0230814)

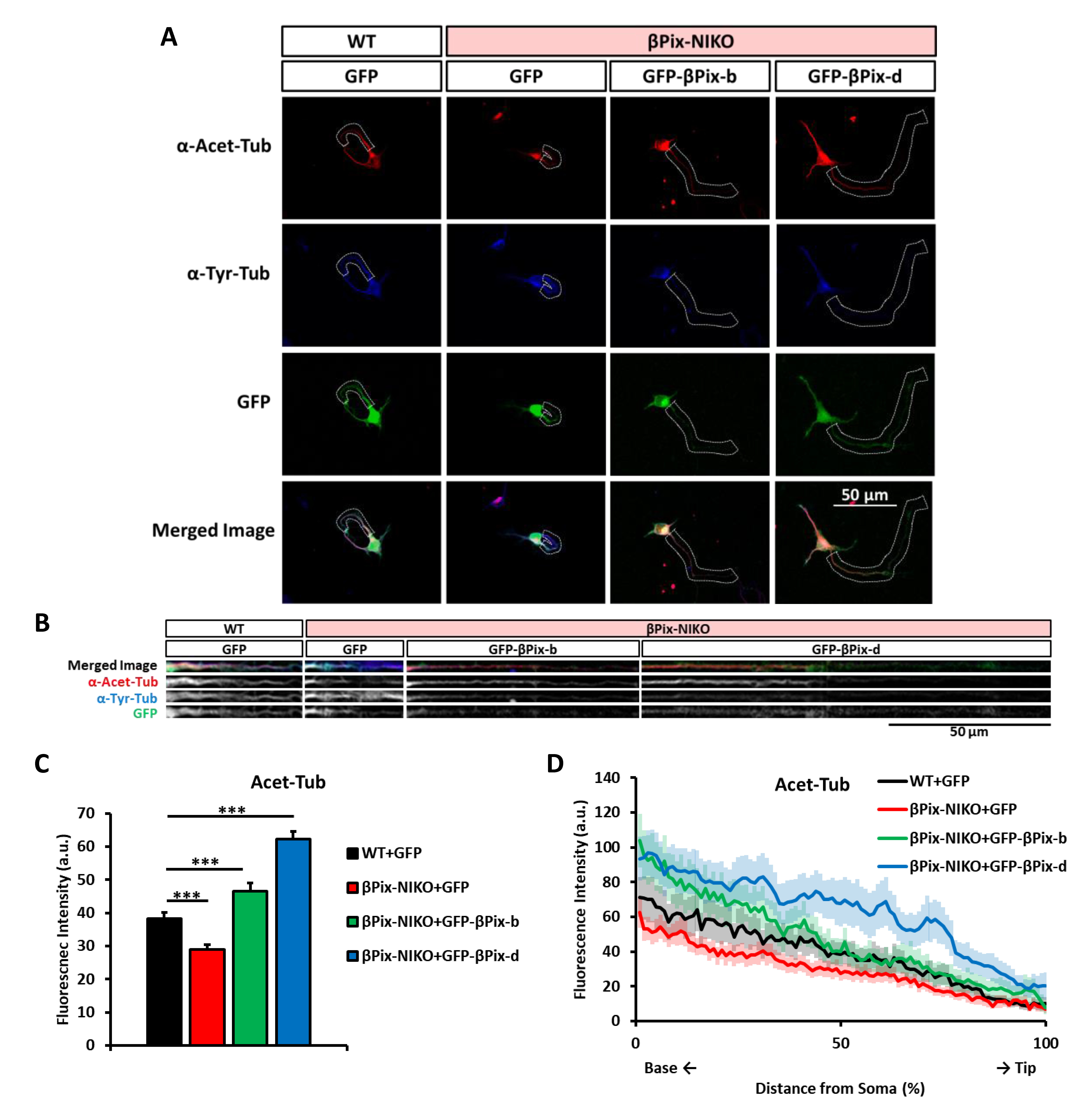

Supplement: S1 Fig — (A) Representative images of WT neurons transfected with GFP and βPix-NIKO neurons transfected with GFP, GFP-βPix-b, or GFP-βPix-d at DIV3, fixed at DIV4, and stained with Acet-Tub antibody (red) and Tyr-Tub antibody (blue). The white dashed lines indicate the longest neurite of the neuron in each group, which is straightened in S1B Fig. (B) The longest neurite of the WT neurons transfected with GFP and βPix-NIKO neurons transfected with GFP, GFP-βPix-b, or GFP-βPix-d from S1A Fig is straightened using ImageJ software. (C) The longest neurite extending from βPix-NIKO neurons has lower mean level of Acet-Tub than that from WT neurons. In βPix-NIKO neurons, expression of βPix-b or βPix-d recovers the decreased mean level of tubulin acetylation and the recovery level of tubulin acetylation was higher than WT neurons. (D) The distribution graph showed that reduced Acet-Tub level in βPix-NIKO neurons was observed, compared with that in WT neuron. The decrease in Acet-Tub was rescued by βPix-b or βPix-d expression in βPix-NIKO neurons. The rescued level was higher than the tubulin acetylation that WT neurons have. n = 18–30 neurons for each group. In (C), *** P < 0.001 by one-way ANOVA followed by post-hoc Tukey’s test. (TIF) [file pone.0230814.s001.tif]

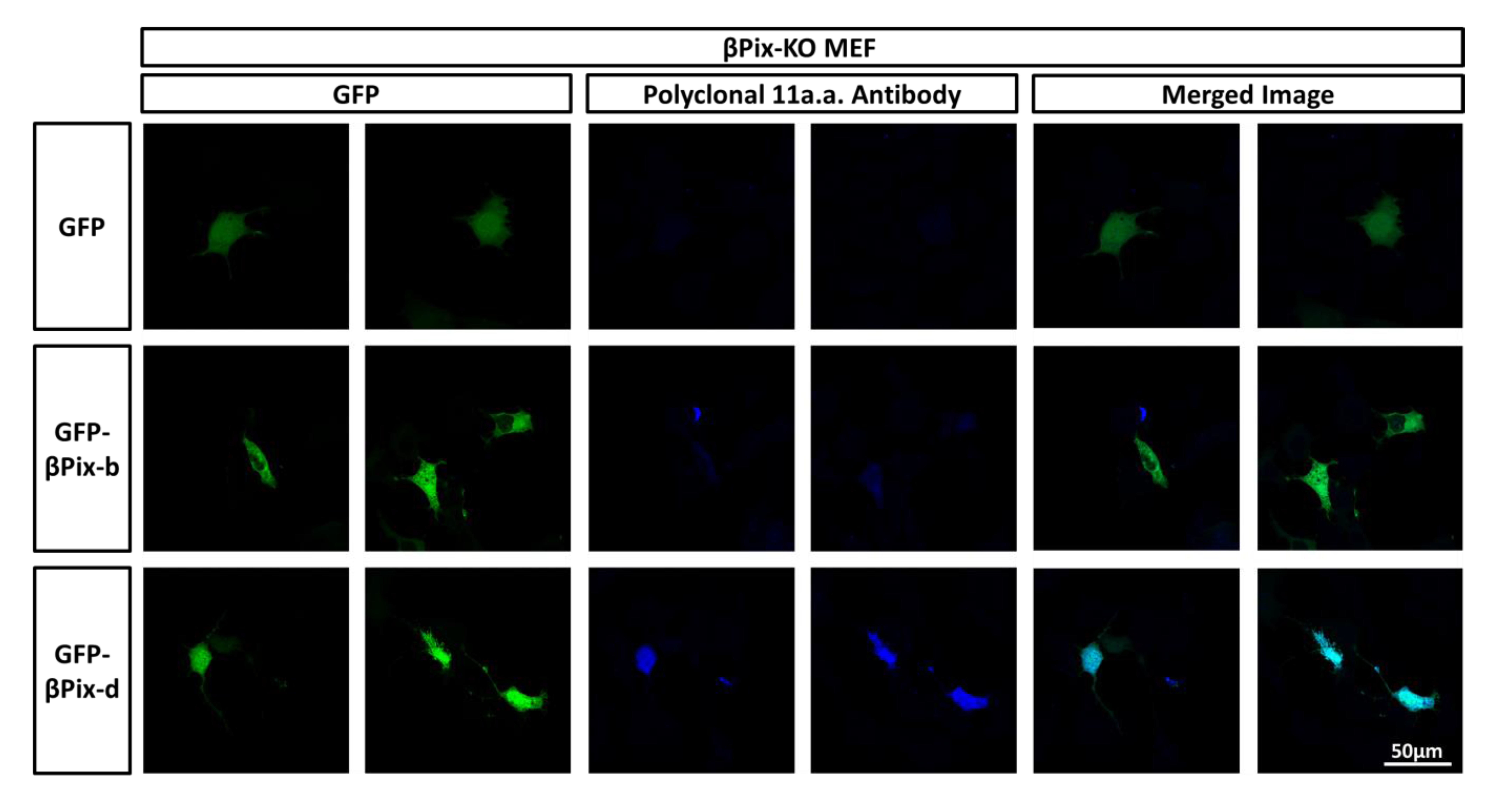

Supplement: S2 Fig — After expression of GFP-βPix-b or GFP-βPix-d in βPix-KO MEFs (35), those MEFs were stained with anti-11 a.a. antibody. By anti-11 a.a. antibody, βPix-b was not detected, but βPix-d was detected. Data from three independent cultures. (TIF) [file pone.0230814.s002.tif]

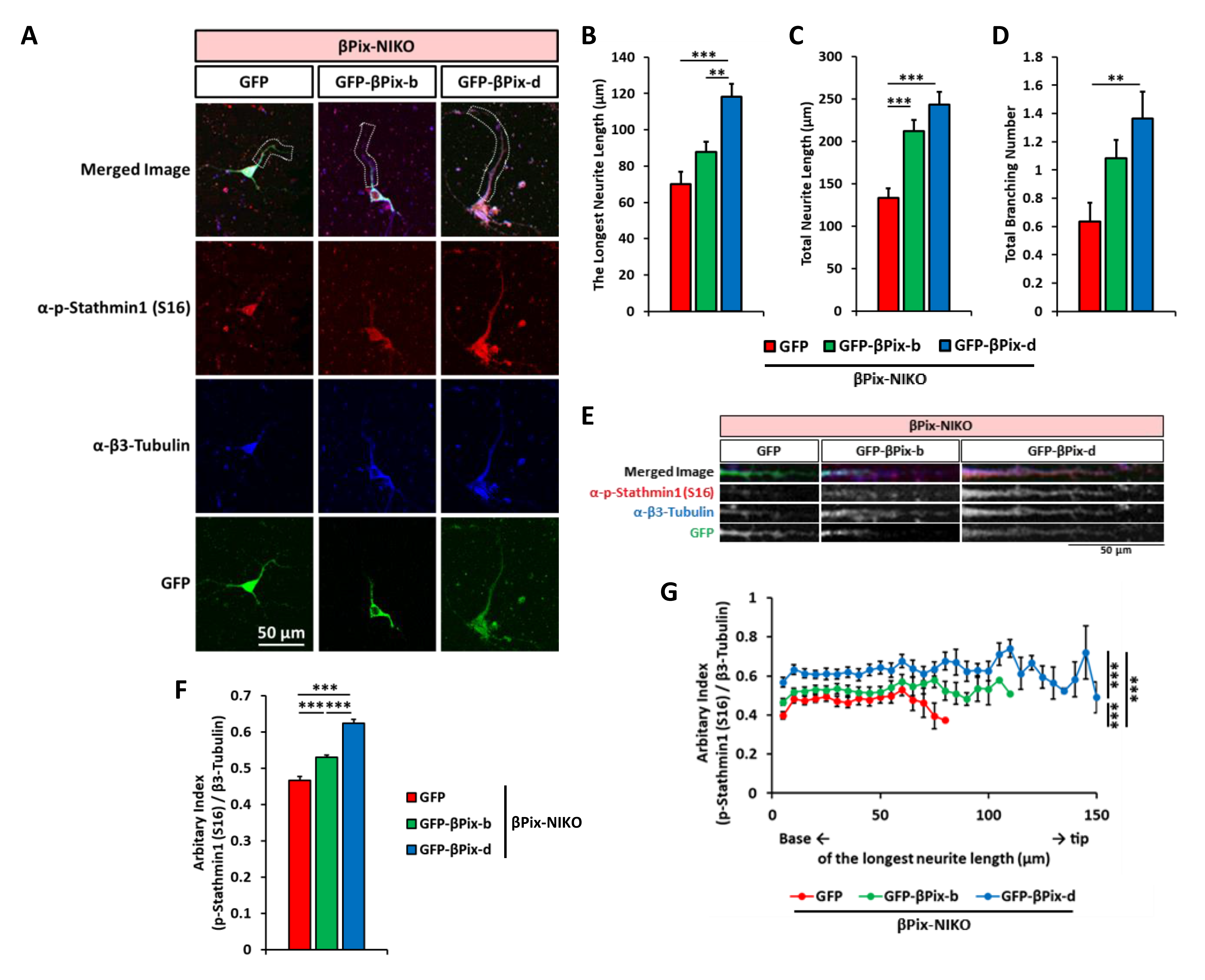

Supplement: S4 Fig — (A) Representative images of βPix-NIKO neurons transfected with GFP, GFP-βPix-b, or GFP-βPix-d at DIV3, fixed at DIV4, and stained with p-Stathmin1 (S16) (red) and β3-tubulin (blue) antibodies. (B) The longest neurite length increases with the expression of GFP-βPix-b or GFP-βPix-d in the βPix-NIKO neurons compared with the GFP-expressing control. (C) Total neurite length is recovered by the expression of GFP-βPix-b or GFP-βPix-d in the βPix-NIKO neurons compared with the GFP-expressing control. (D) Total branching number is recovered by the expression of GFP-βPix-b or GFP-βPix-d in the βPix-NIKO neurons compared with the GFP-expressing control. (E) The longest neurites shown in (A) are straightened using ImageJ software. (F) Phosphorylation levels of Stathmin1 at Ser16 in the longest neurite are recovered by expressing GFP-βPix-b or GFP-βPix-d in βPix-NIKO neurons compared with the GFP-expressing control. The phosphorylated Stathmin1 levels are normalized to β3-tubulin. (G) The phosphorylation of Stathmin1 at Ser16 is recovered by expressing GFP-βPix-b or GFP-βPix-d compared with the GFP-expressing control along the longest neurite extending from the βPix-NIKO neurons. n = 61–82 neurons per group from three independent cultures. In (B)–(D), (G) and (H), * P < 0.01 and *** P < 0.001 by one-way ANOVA followed by post-hoc Tukey’s test. (TIF) [file pone.0230814.s004.tif]
