# Supplementary material for: βPix-d promotes tubulin acetylation and neurite outgrowth through a PAK/Stathmin1 signaling pathway
Source: PLoS One. 2020 Apr 6;15(4):e0230814. doi: 10.1371/journal.pone.0230814 (PMC7135283; doi:10.1371/journal.pone.0230814)
Supplement: S6 Fig — (PDF) [file pone.0230814.s006.pdf]

C

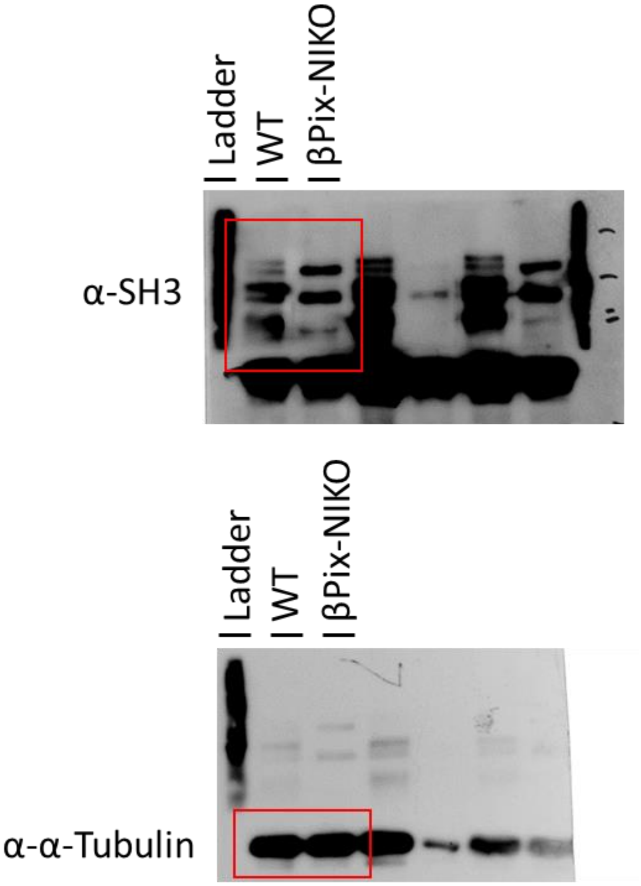

Fig 1C raw image, Kwon et al.

E

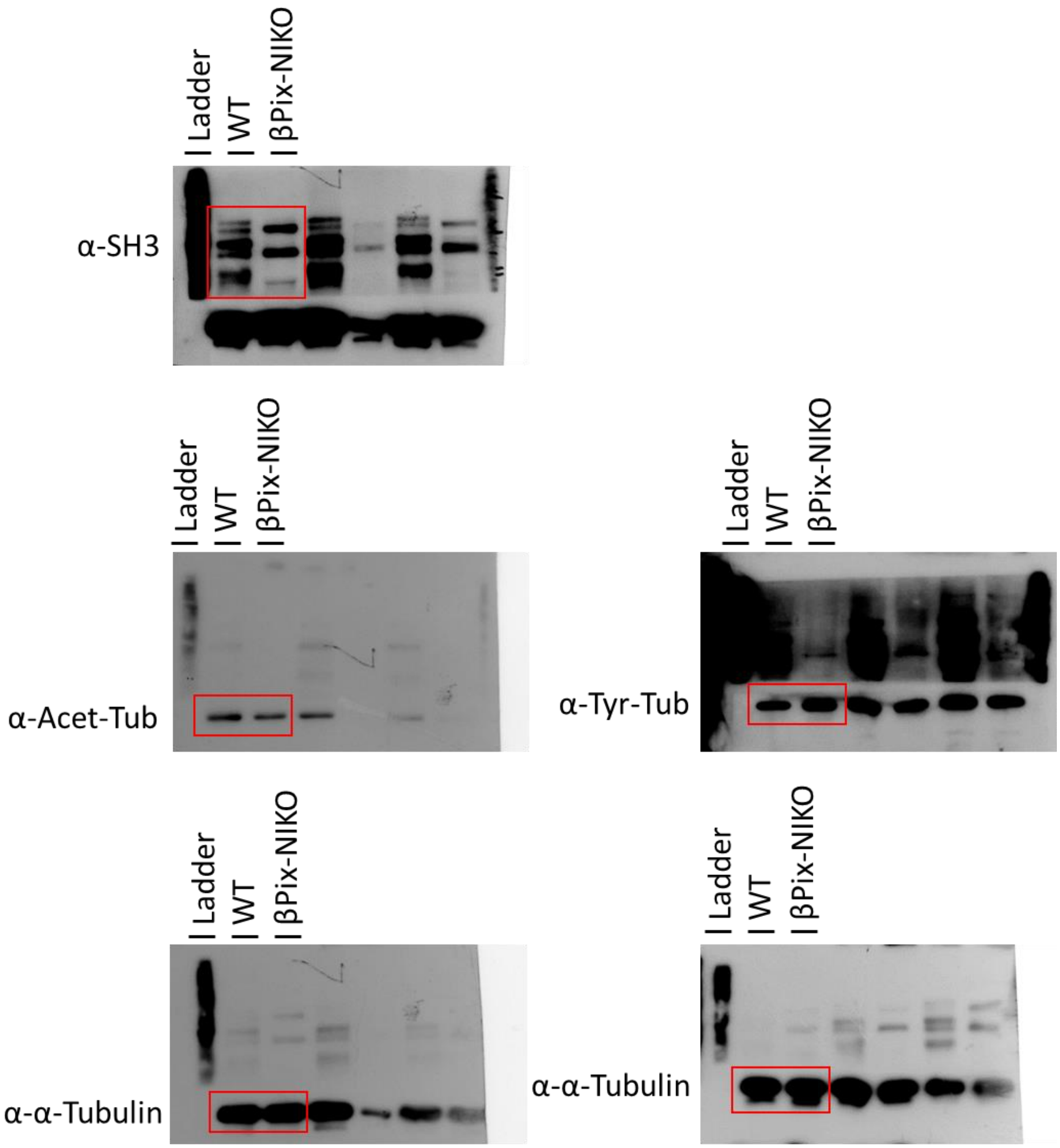

Fig 2E raw image, Kwon et al.

A

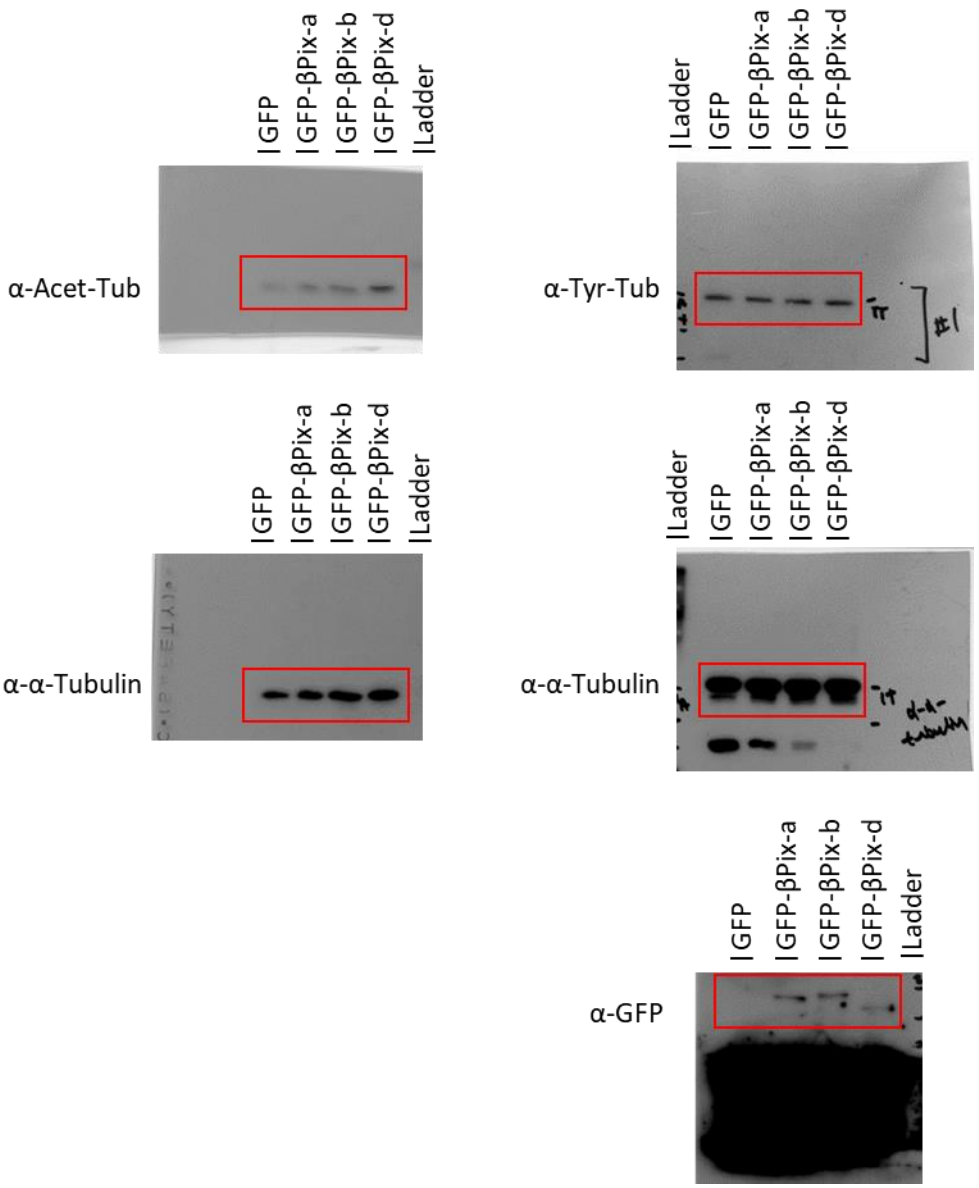

Fig 4A raw image, Kwon et al.

H

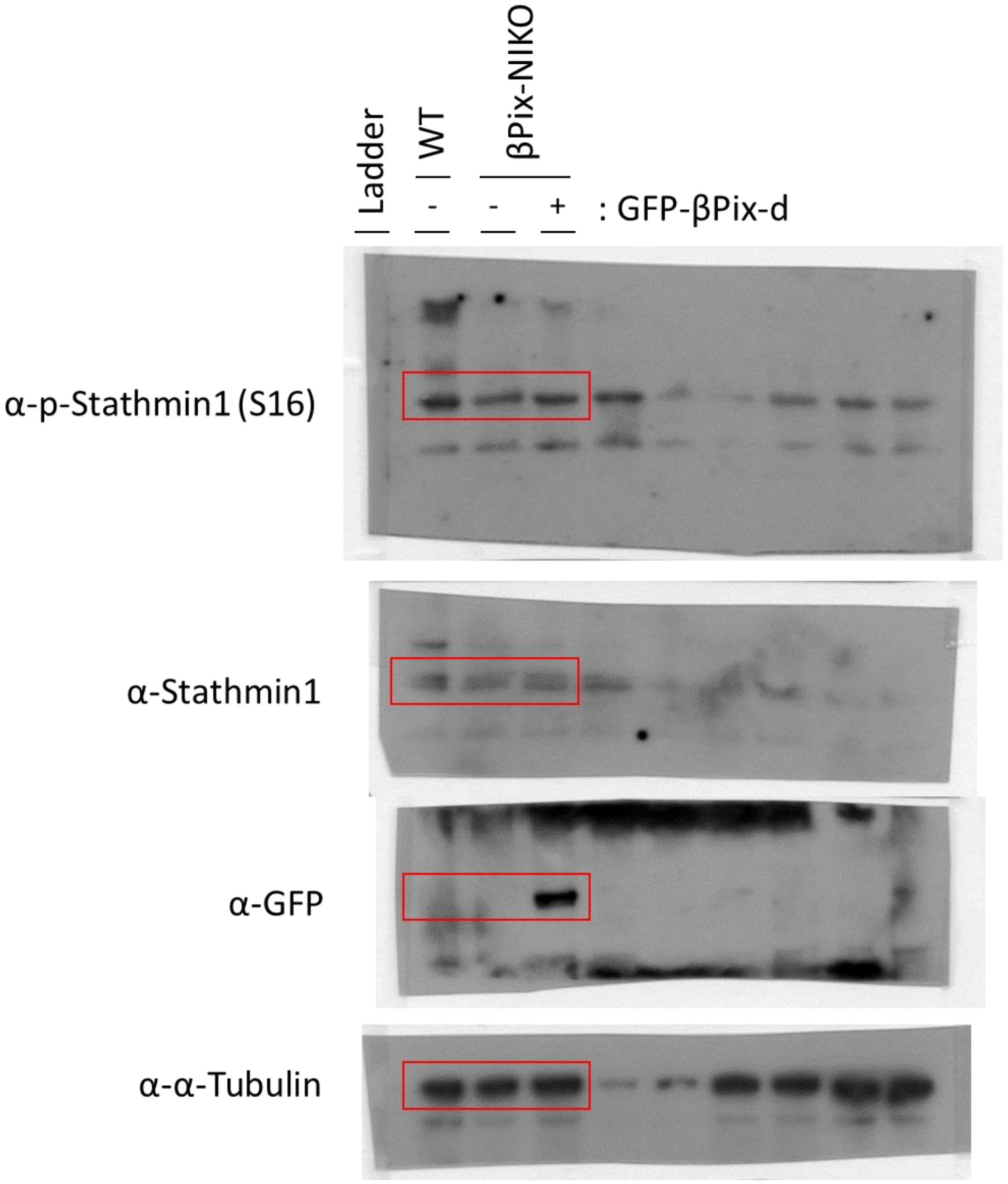

Fig 5H raw image, Kwon et al.
